# LipiDecipher: A Structure-Oriented Analytical Framework for Interpretable Clinical Lipidomics

**DOI:** 10.3390/metabo16070494

**Published:** 2026-07-13

**Authors:** Anliang Huang, Yunshu Zhang, Baoning Wu, Tingting Bai, Xiaoyang Yuan, Dong Shang, Shurong Ma, Rihong Huang, Peiyuan Yin

**Affiliations:** 1 Laboratory of Integrative Medicine, The First Affiliated Hospital of Dalian Medical University, Dalian 116000, China; aaronhwang8720@163.com (A.H.); zys1986062186@163.com (Y.Z.); shangdong@dmu.edu.cn (D.S.); mashurong@dmu.edu.cn (S.M.); 2 School of Integrated Traditional Chinese and Western Medicine, Dalian Medical University, Dalian 116044, China; 3 Department of General Surgery, The First Affiliated Hospital of Dalian Medical University, Dalian 116000, China; 4 General Surgery, Dalian Third People’s Hospital Affiliated to Dalian University of Technology, Dalian 116000, China; wbn121488@163.com; 5 Department of Cardiology, The First Affiliated Hospital of Dalian Medical University, Dalian 116000, China; baitingting-1985@163.com (T.B.); sunday999_1999@163.com (X.Y.)

**Keywords:** lipidomics, myocardial infarction, computational biology, bioinformatics, structure–function relationship, lipid deconstruction, biomarker discovery

## Abstract

Background: Clinical lipidomics can capture disease-associated molecular alterations at high resolution, yet translating complex lipid species data into interpretable biological insight remains challenging. Existing workflows often emphasize statistical discrimination while underutilizing the structural information embedded in lipid species. To address this gap, we developed LipiDecipher, a structure-oriented analytical framework designed to summarize lipidomic alterations into interpretable structural patterns and to provide database-supported biological contextualization. Methods: LipiDecipher integrates differential lipid analysis, structure-resolved summarization, multivariate discrimination, and knowledge-based lipid-to-protein/pathway contextualization. We applied this framework to a retrospective serum lipidomics dataset comprising healthy controls and patients with acute myocardial infarction or post-PCI recurrent myocardial infarction. To improve transparency and robustness, the revised analysis includes sex-disaggregated reporting, covariate-adjusted sensitivity analyses for sex and age, and internal separation stability assessment of category-specific LDA projections through resampling-based feature stability analysis, repeated cross-validation, and permutation testing. Results: The framework identified distinct lipid alterations across study groups, including changes in phosphatidylinositols, ceramides, and triglyceride remodeling patterns. These alterations became more interpretable when summarized at the structural level, including lipid class composition, acyl-chain length, and degree of unsaturation. Internal discrimination analyses suggested separability between groups, while repeated resampling highlighted a subset of recurrently selected lipid features. Knowledge-based mapping prioritized lipid-associated biological contexts related to glycerophospholipid metabolism, sphingolipid metabolism, membrane remodeling, inflammatory signaling, and energy-related processes. Importantly, these protein- and pathway-level outputs are presented as database-supported hypotheses rather than direct evidence of target engagement or pathway activation in the studied cohort. Conclusions: LipiDecipher provides a structure-oriented and interpretation-focused framework for clinical lipidomics. In a retrospective acute myocardial infarction cohort, it enabled the prioritization of candidate lipid signatures and biologically plausible hypotheses from complex lipidomic data. These findings support its use as a hypothesis-generating analytical tool, while external validation and experimental follow-up remain necessary before mechanistic or clinical claims can be established.

## 1. Introduction

Lipids are fundamental biological molecules essential for cellular structure, energy homeostasis, and signal transduction. While conventional clinical assays measuring total triglycerides and cholesterol serve as primary predictors for cardiovascular events, their diagnostic specificity and early-prediction sensitivity are limited [1,2]. The advent of high-throughput mass spectrometry has enabled the deep structural annotation of the plasma lipidome at individual acyl-chain resolution, significantly enhancing prognostic accuracy for complex diseases [3]. In the post-genomic era, clinical lipidomics, driven by specific clinical questions and the complexity of patient samples, promises to revolutionize early diagnosis, precision medicine, and therapeutic strategies [4,5,6,7].

Despite our ability to measure thousands of lipid species, the sheer diversity and structural complexity of the lipidome present formidable analytical and interpretive challenges. Current research is often confined to analyzing individual lipid classes, failing to capture the global, systems-level picture. In other omics disciplines, functional interpretation is greatly facilitated by well-established frameworks like Gene Ontology [8] (GO) and pathway enrichment analysis (e.g., Kyoto Encyclopedia of Genes and Genomes [9,10,11] (KEGG), which effectively translate vast datasets into biological insights. However, the lipidomics field currently lacks a comparably mature analytical paradigm, creating a significant bottleneck.

This gap stems from a fundamental mismatch between generic analytical logic and the unique nature of lipids. The biological function of a lipid is intrinsically tied to its detailed chemical structure, yet most analytical workflows, particularly those adapted from general metabolomics, fail to account for this. They often overlook the critical fact that subtle structural variations—such as differences in fatty acyl chain length or the number of double bonds—can lead to vastly different, or even opposing, biological activities within the same lipid class. This limitation often restricts functional analysis to a superficial level and may obscure structure-associated patterns that could inform downstream biological interpretation. To unlock the full clinical potential of lipidomics, a new analytical strategy is urgently needed—one that can systematically organize lipid structural features into interpretable analytical layers and support more biologically informed, hypothesis-generating interpretation.

**Figure 1:**
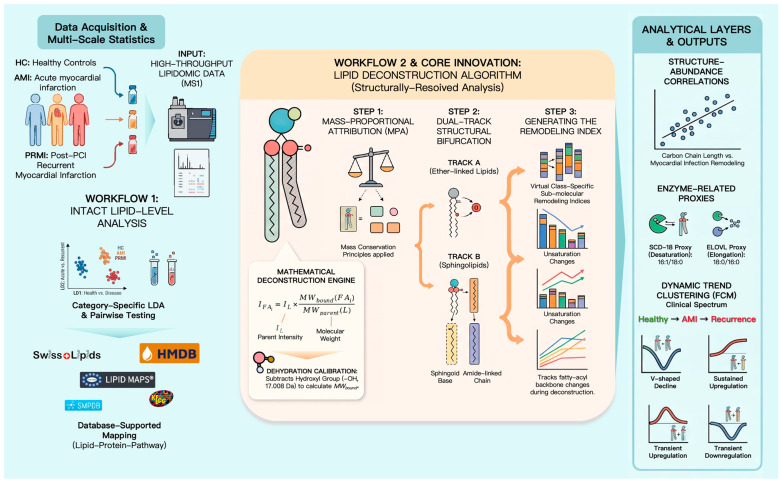
Overall flowchart for LipiDecipher.

To address this challenge, we developed LipiDecipher, a comprehensive and modular analytical framework designed to navigate the complexities of lipidomics data. The overall workflow, from data acquisition to biological interpretation, is schematically illustrated in Figure 1. LipiDecipher integrates multi-scale statistical analyses with a structure-centric strategy to facilitate biologically informed interpretation. The first arm of this strategy, Workflow 1: Intact Lipid-Level Analysis, focuses on intact lipid molecules and extends conventional statistical analysis by incorporating a “lipid–protein–pathway” mapping step for biological contextualization. This approach links lipid structures of interest to curated protein- and pathway-associated knowledge, aiming to prioritize biologically plausible hypotheses rather than to claim direct target engagement. The second arm, Workflow 2: Structurally-Resolved Analysis, examines sub-molecular features through a lipid deconstruction module. Crucially, unlike conventional gas chromatography–mass spectrometry (GC-MS) following chemical hydrolysis, which inevitably destroys the chemical connection between polar headgroups and hydrophobic tails, LipiDecipher mathematically projects intact lipid abundances into class-specific sub-molecular chambers. These deconstructed values do not represent the absolute physical concentrations of free fatty acids, but rather serve as virtual sub-molecular remodeling indices, also referred to as class-specific acyl partitioning indices. This approach preserves the crucial metabolic context of the parent lipid class while uncovering systematic fatty acyl remodeling patterns and their associations with clinical phenotypes. Through this dual-workflow design, LipiDecipher is presented as a reproducible and extensible analytical framework. To facilitate transparency and reuse, we provide a detailed case study and open-source R code (available at https://github.com/AaronHwang8720/LipiDecipher (accessed on 7 July 2026)).

## 2. Methods

### 2.1. Clinical Cohort and Sample Information

This study enrolled a retrospective clinical cohort comprising three groups of subjects: healthy controls (HC, *n* = 50), patients with acute myocardial infarction (AMI, *n* = 50), and patients with post-PCI recurrent myocardial infarction (PRMI, *n* = 35). The study design and reporting conform to the Strengthening the Reporting of Observational Studies in Epidemiology (STROBE) guidelines for cohort studies. The completed STROBE checklist is provided as Supplementary Materials. Baseline demographic characteristics of the cohort, including sex and age, are summarized in Table 1 and Supplementary Table S1. Baseline demographic variables were compared across study groups to assess potential imbalance. Sex distribution was evaluated using the chi-square test or Fisher’s exact test where appropriate, and age was compared using one-way analysis of variance. Pairwise comparisons were additionally performed to identify the source of any group differences. To assess the potential influence of baseline demographic imbalance on lipidomic associations, sensitivity analyses were performed using linear models. For each lipid feature, the unadjusted model included study group as the main predictor, whereas adjusted models additionally included sex alone or sex plus age as covariates. Directional consistency of group-effect estimates between unadjusted and adjusted models was assessed for the AMI vs. HC and PRMI vs. HC comparisons. Sex-stratified summaries were also generated for the major lipid features and are provided in Supplementary Table S2. A graphical summary of covariate-adjusted sensitivity analyses is provided in Supplementary Figure S2, and the corresponding summary statistics are provided in Supplementary Table S3.

### 2.2. UHPLC-MS/MS Lipidomics Analysis

#### 2.2.1. Sample Preparation and Lipid Extraction

For the untargeted lipidomic profiling of the hydrophobic non-polar fractions (Methods M4 and M5), a modified methyl tert-butyl ether (MTBE) liquid–liquid extraction protocol was executed to preserve structural integrity and prevent degradation. Thawed serum aliquots (20 μL) were transferred into 1.5 mL Eppendorf tubes and spiked with 120 μL of ice-cold mass-spectrometry-grade methanol containing a custom-formulated mix of named stable isotope-labeled extraction internal standards. This extraction carrier included: LysoPE (18:1-d7), LysoPC (18:1-d7), SM (d18:1/18:1-d9), PC (15:0/18:1-d7), PE (15:0/18:1-d7), ChE (18:1-d7), TG (15:0/18:1-d7/15:0) and PG (15:0/18:1-d7). The mixture was vortexed aggressively for 180 s to ensure complete protein precipitation and membrane lysis. Subsequently, 360 μL of chromatographic-grade MTBE and 100 μL of Milli-Q ultrapure water were introduced to induce biphasic separation. The homogenate was shaken continuously for 10 min and allowed to stand at 4 °C for 10 min to reach phase equilibrium. Following centrifugation at 15,000 *g* for 15 min at 4 °C, a 300 μL aliquot of the upper lipophilic organic phase was harvested, transferred to a clean tube, and evaporated to absolute dryness using a vacuum centrifugal concentrator.

Prior to instrumental injection, the dry lipid residue was reconstituted in 150 μL of an acetonitrile-isopropanol mixture (1:1, *v*/*v*) fortified with a commercial SPLASH^®^ LIPIDOMIX^®^ mass-spectrometry internal standard mixture (Avanti Polar Lipids, Alabaster, AL, USA). The precise extraction concentrations, chemical formulations, and compound-specific diagnostic properties for the entire internal standard panel are explicitly tabulated in Supplementary Table S5. Quality control (QC) samples were prepared by pooling equal volume fractions from all experimental cohorts and processed synchronously alongside individual patient samples.

#### 2.2.2. UHPLC-HRMS Untargeted Lipidomics Analysis

Untargeted analysis was conducted on a UPLC-HRMS system, consisting of a Thermo Scientific Ultimate™ 3000 UPLC system coupled to a Thermo Scientific Q Exactive™ Quadrupole-Orbitrap high-resolution mass spectrometer (Thermo Fisher Scientific, Waltham, MA, USA). Chromatographic separation was achieved using an Accucore C30 core–shell column. The mobile phase consisted of (A) 60% acetonitrile in water and (B) 90% isopropanol/10% acetonitrile, both containing ammonium formate and 0.1% formic acid. The gradient elution program was as follows: initial 10% B, ramped linearly to 50% B in 5 min, then further ramped to 100% B over 23 min.

The mass spectrometer was equipped with a heated electrospray ionization (H-ESI) source, and data were acquired in both positive and negative ionization modes. Ionization parameters were set as follows: sheath gas flow rate, 45 arb; auxiliary gas flow rate, 10 arb; capillary temperature, 320 °C; heater temperature, 355 °C; S-Lens RF level, 55. Data were collected in full scan mode.

#### 2.2.3. Lipidomics Data Preprocessing and Multi-Stage Filtering Cascade

The raw mass spectrometry profile data were parsed, peak-picked, and aligned using LipidSearch software version 4.1 (Thermo Fisher Scientific, Waltham, MA, USA). Precursor and product ion discovery was executed by matching experimentally observed MS/MS fragments against in silico predicted fragmentation spectra of diverse lipid classes, including phospholipids, neutral glycerolipids, sphingolipids, neutral glycosphingolipids, glycosphingolipids, steroids, and fatty esters. Search constraints enforced a strict mass accuracy threshold of 5 ppm for MS1 precursor ions and 5 mDa for MS2 product ions, utilizing an MS/MS similarity score threshold cutoff of 5. The tracked ionization adduct species included $[M+H{]}^{+},[M+Na{]}^{+},\;\mathrm{and}\;[M+NH_{4}{]}^{+}$ in positive mode, alongside $[M-H{]}^{-},[M+FA-H{]}^{-},\;\mathrm{and}\;[M+Ac-H{]}^{-}$ in negative mode. To eliminate chemical noise and false positives, all automated identifications underwent manual one-by-one curation, assessing isotopic peak shapes, adduct ion co-elution behaviors, neutral loss diagnostic patterns, and chromatographic retention behavior alignment rules.

To satisfy reporting standards regarding data filtering and robustness, the final lipid feature space was derived via a multi-stage filtering cascade: (1) Initial Raw Peak Catching: A total of 436 raw lipid features were originally detected across the analytical run. (2) Missing-Value Quality Filtering: Features exhibiting a missing-value rate exceeding 50% within the consecutive QC sample injections or exceeding 20% across all individual biological cohorts were discarded, eliminating 17 low-confidence features and yielding 419 lipids. (3) Analytical Stability Filtering: Features exhibiting poor reproducibility, characterized by a coefficient of variation (CV) exceeding 30% across the repeated pool QC injections, were pruned out. This stage removed another 6 unstable features, successfully retaining 413 highly reproducible, robust lipid species for downstream analysis.

Residual isolated missing values within the final 413 feature matrix were imputed using the k-Nearest Neighbors (kNN) algorithm based on the Euclidean distance metrics of localized feature blocks.

#### 2.2.4. Metabolomics Standards Initiative (MSI) Structural Taxonomy Mapping

To provide transparent methodological documentation and clarify the experimental boundaries of the structure-oriented deconstruction module, all 413 validated lipid features were explicitly mapped onto the structural taxonomy proposed by the Metabolomics Standards Initiative (MSI) reporting standards. This systematic classification strictly bifurcated our data into experimentally supported chains and computationally inferred summaries: (1) MSI Level 2 (Highly Resolved Molecular Species; Class Levels A and B): Assigned exclusively to lipid species whose high-resolution secondary MS/MS spectra yielded distinct, unambiguous diagnostic fragment fingerprints detailing individual fatty acyl chain compositions (e.g., resolving a phosphatidylcholine down to PC (16:0_18:1)). Class Level A signifies unequivocal structural confirmation where both distinct chains possess concrete product ion fragments, whereas Class Level B indicates high-confidence structures where localized matrix rule-matching supports chain positioning. (2) MSI Level 3 (Sum-Composition Level; Class Levels C and D): Assigned to features where exact precursor mass (*m*/*z*) and accurate MS1 isotopic profiles established the chemical formula, but the corresponding MS/MS spectra lacked distinctive fragment signatures to resolve individual acyl chains (e.g., remaining trapped at the total carbon/unsaturation sum-composition level, such as PC (34:1)). Class Level C defines sum-level species with robust baseline peak shapes, while Class Level D traps emerging low-abundance boundaries.

The comprehensive architectural breakdown, including the exact precursor mass, ionization adduct parameters, MSI identification levels, and specific Class Level labels for all 413 monitored species, is cataloged in Supplementary Table S4, ensuring complete structural visibility prior to data deconstruction calculations.

### 2.3. Overview of the LipiDecipher Framework

LipiDecipher is an interpretation-oriented workflow composed of four analytical layers: (1) statistical prioritization of differential lipid features across clinical groups; (2) structure-oriented summarization of lipid alterations at the levels of lipid class, carbon number, and unsaturation; (3) knowledge-based biological contextualization through database-supported mapping of lipid species to lipid-associated proteins and pathways; and (4) robustness assessment, including covariate-adjusted sensitivity analyses and internal evaluation of discriminant robustness through resampling-based feature stability analysis, repeated cross-validation, and permutation testing. In this framework, protein- and pathway-level outputs are not interpreted as direct evidence of molecular binding, target activation, pathway activation, or causal mechanism. Instead, they are used to contextualize differential lipid signatures within curated biochemical and biological knowledge and to prioritize hypotheses for downstream validation.

### 2.4. Sub-Molecular Lipid Deconstruction and Adaptively Calibrated Mass-Proportional Attribution

To bypass the generic “intact-molecule” analysis bottleneck and extract sub-molecular architectural variations, LipiDecipher implements an infrastructure-level data deconstruction engine. Rather than directly treating intact lipid species as isolated, unresolvable features, this module mathematically projects and partitions the semi-quantitative instrumental response (MS1 peak area) of an intact parent lipid molecule (L) into its constituent individual combination blocks (e.g., standard ester-bound acyl chains, ether/alkyl linkages, or sphingoid base backbones) based on strict mass conservation principles.

To prevent systemic signal inflation—wherein assigning the total parental abundance directly to each cleaved chain would artificially inflate the total system signal to 200% for diacyl phospholipids or 300% for triacylglycerols (TG)—the framework introduces a strict adaptively calibrated mass-proportional attribution (MPA) algorithm.

### 2.5. Mathematical Formulation for Standard Ester-Bound Acyl Chains

For a standard intact diacyl or triacyl lipid molecule (L) containing n symmetric or asymmetric ester-bound fatty acyl chains, the chemical formula of any given individual cleaved free fatty acid ($FA_{i}$) is parameter-defined by its carbon atom number ($C_{i}$) and double bond count ($DB_{i}$). Its theoretical physical molecular weight ($MW_{free}(FA_{i})$) is rigorously calculated via the following equation:

$$MW_{free}(FA_{i})=12.011\times C_{i}+1.008\times (2C_{i}-2DB_{i})+15.999\times 2$$

Crucially, when esterified onto a glycerol backbone, each acyl chain inevitably undergoes a dehydration condensation reaction, forfeiting a hydroxyl group (−OH,17.008 Da). To balance the structural mass matrix, the net physical weight of the bound acyl residue ($MW_{bound}(FA_{i})$) is actively calibrated as:

$$\begin{array}{c} MW_{bound}(FA_{i})=MW_{free}(FA_{i})-17.008 \end{array}$$

### 2.6. Dual-Track Regular-Expression Partitioning for Special Linkages

To eliminate over-simplified “one-size-fits-all” calculations across structurally highly heterogeneous lipid classes, LipiDecipher deploys a regular-expression-driven dual-track bifurcation pipeline to automatically isolate ether-linked fatty chains and sphingoid backbones:

Track A: Ether-linked Phospholipids (Alkyl and Plasmalogen Species)

For lipid species marked with an O- (alkyl, suffix e) or P- (alk-1-enyl, plasmalogen, suffix p) designation, the functional group attached to the glycerol backbone contains a single ether oxygen bridge instead of a standard ester carbonyl group. For Alkyl ether chains (suffix e), the absolute mass of the bound residue is calculated by stripping one oxygen atom from the fatty acid matrix:

$$\begin{array}{c} MW_{bound}(FA_{i,alkyl})=12.011\times C_{i}+1.008\times (2C_{i}+2-2DB_{i})+15.999-17.008 \end{array}$$

For Alk-1-enyl ether (Plasmalogen) chains (suffix p), the calibration is mathematically established as:

$$\begin{array}{c} MW_{bound}(FA_{i,plasmalogen})=12.011\times C_{i}+1.008\times (2C_{i}-2DB_{i})+15.999-17.008 \end{array}$$

Track B: Sphingolipids (Ceramides and Sphingomyelins)

For sphingolipids containing a sphingoid long-chain base (LCB, prefixed with d for dihydroxy or t for trihydroxy, e.g., d18:1) and an amide-linked fatty acyl chain, LipiDecipher parses them as two independent chemical entities. To mirror the steady-state composition balance in sub-molecular chambers, their mass fractional proportions are defined via Sub-molecular Clipping Weights (ω). For any structural moiety j bound within a parent sphingolipid, its clipping weight is normalized against the total calculated moiety weight sum of the parental molecule:

$$\begin{array}{c} \omega_{j}=\frac{MW_{bound}(Moiety_{j})}{\sum_{k=1}^{m}MW_{bound}(Moiety_{k})} \end{array}$$

### 2.7. Mass-Conservation Signal Deconvolution

Once the bound weights or clipping weights of all structural moieties within a parent lipid molecule (L) are derived, the total quantitative instrumental intensity of the intact lipid ($I_{L}$) is proportionally partitioned. The mathematically attributed virtual abundance of a single deconstructed moiety ($I_{FA_{i}}$) is calculated via Equation

$$I_{FA_{i}}=I_{L}\times \frac{MW_{bound}(FA_{i})}{MW_{parent}(L)}$$

where $MW_{parent}(L)$ represents the exact mass (EXACT_MASS) of the intact parent lipid molecule. Following the intra-molecular intensity division, the calculated fragments of identical sub-molecular moieties are aggregated and aggregated independently within each specific lipid Main Class (C) to yield the unified Virtual Class-Specific Sub-molecular Remodeling Index ($I_{total}(FA,C)$):

$$\begin{array}{c} I_{total}(FA,C)=\sum_{j\in C}I_{FA_{i}}(L_{j}) \end{array}$$

These deconstructed summaries do not represent the direct physical absolute concentrations of free circulating fatty acids, but rather serve as class-contextualized indices tracing systematic structural remodeling patterns across independent clinical cohorts.

### 2.8. Structure–Abundance Correlation Analysis

To investigate whether lipid metabolic remodeling during disease follows specific structural rules, we quantified the relationship between changes in lipid abundance and fatty acyl chain structural parameters. Specifically, we calculated the log2 fold change (log2FC) for each fatty acyl chain between different clinical comparison groups and used linear regression models to test the correlation of log2FC with the number of carbon atoms and double bonds, respectively. Significant positive or negative correlations may be compatible with systematic structure-associated remodeling and can generate hypotheses regarding upstream processes such as elongation or desaturation; however, they do not directly measure enzyme activity.

### 2.9. Proxy-Based Assessment of Fatty Acid Remodeling Ratios

To evaluate changes in key fatty acid metabolic pathways (e.g., elongation and desaturation), we employed a dual analysis strategy. This approach combined product-to-substrate ratio proxies for selected lipid remodeling patterns with a systemic assessment of these pathways across the lipidome using structure–abundance correlation analysis.

### 2.10. Product-to-Substrate Ratio Analysis as Enzyme-Related Proxies

To estimate lipid remodeling patterns, product-to-substrate ratios were calculated as enzyme-related proxies for selected metabolic pathways. These ratios included SCD1 (16:1/16:0, 18:1/18:0), FADS1 (20:4/20:3), FADS2 (18:3/18:2), and a general elongase proxy (18:0/16:0). These calculated ratios represent descriptive metabolic readouts of steady-state lipid composition rather than direct measurements of active enzymatic rates. The statistical significance of the differences in these ratios between clinical groups was assessed using the non-parametric Wilcoxon rank-sum test due to the non-normal distribution profiles of the calculated sub-molecular product-to-substrate ratios, with the resulting *p*-values controlled for multiple comparisons via the Benjamini–Hochberg FDR procedure.

### 2.11. Systemic Assessment: Structure–Abundance Correlation Analysis

To systematically test for global trends in fatty acid remodeling, we performed a structure–abundance correlation analysis. This method quantifies the linear relationship between a lipid’s structural attributes and its magnitude of abundance change between comparison groups.

First, for each lipid molecule (L), the log2FC was calculated for each group comparison (e.g., AMI vs. HC) using the formula:

$$\begin{array}{l} {\log_{2}FC}_{L}=\log_{2}\left(\frac{{\bar{I}}_{L,AMI}}{{\bar{I}}_{L,HC}}\right) \end{array}$$

where ${\bar{I}}_{L,AMI}$ and ${\bar{I}}_{L,HC}$ represent the mean abundance of lipid L in the AMI and HC groups, respectively. Concurrently, two key structural parameters were extracted from each lipid’s annotation—$C_{L}$: The total number of carbon atoms in the fatty acyl chains. ${DB}_{L}$: The total number of double bonds in the fatty acyl chains.

Next, two independent simple linear regression models were constructed to assess the relationship between these structural parameters and the calculated log2FC.

Model for Carbon Chain Length: To model the dependency of abundance change on the total number of carbons, the following equation was used:

(1) $$\begin{array}{l} {\log_{2}FC}_{L}=\beta_{0}+\beta_{1}\times C_{L}+\varepsilon \end{array}$$

Model for Double Bond Count: Similarly, to model the dependency on the total number of double bonds, the following equation was used:

(2) $$\begin{array}{l} {\log_{2}FC}_{L}=\beta_{0}+\beta_{1}\times {DB}_{L}+\varepsilon \end{array}$$

For each model, the statistical significance of the relationship was determined by performing a t-test on the slope coefficient (β_1_), under the null hypothesis that β_1_ = 0. The Pearson’s correlation coefficient (r) was also calculated to measure the strength and direction of the linear association. The analysis was visualized using scatter plots displaying the data points, the linear regression fit line, and its 95% confidence interval.

### 2.12. Statistical Analysis and Visualization

#### Multivariate Pattern Recognition

To assess the global distribution patterns and multi-dimensional variance of the entire cohort, unsupervised Principal Component Analysis (PCA) was first employed to visualize the spontaneous spatial clustering of biological samples and pooled QC replicates. To further extract and prioritize the specific lipid feature blocks driving the contrasts among clinical phenotypes, supervised Linear Discriminant Analysis (LDA) was performed independently within each individual lipid category (ST, SP, GP, GL). This category-specific approach mapped within-cohort descriptive discriminant structures and highlighted major features based on the magnitude of their canonical loading coefficients. The apparent classification cross-tabulations were summarized strictly for descriptive visualization purposes within this study collective. To construct a unified geometric subspace for the descriptive visualization of the cohort, initial data preprocessing stages—including missing-value kNN imputation, variance-based feature filtering, and canonical matrix scaling—were executed globally across the complete dataset prior to dimensional projection, ensuring identical spatial alignment constraints. Concurrently, to evaluate whether the resulting descriptive discriminant structures were robust against sample perturbations rather than artifacts of random label structures, downstream validation loops—including 100-iteration stratified 80% subsampling, repeated stratified 5-fold cross-validation, and 200-cycle random label permutation testing—were subsequently executed as internal consistency metrics.

### 2.13. Differential Lipid Analysis and Multiple Testing Correction

Univariate significance screening was executed to quantify lipid alterations across the critical clinical transitions. Global statistical screening across the three experimental arms (HC, AMI, PRMI) was performed using a one-way analysis of variance (ANOVA) followed downstream by Dunnett’s post hoc tests to specifically isolate pairwise directional differences (AMI vs. HC and PRMI vs. HC).

To rigorously control for Type I errors and address the false-positive inflation inherent to high-dimensional lipidomics datasets, the Benjamini–Hochberg false discovery rate (FDR) correction procedure was systematically and consistently applied across the entire 413 lipid features space simultaneously. An adjusted *p*-value (Adj. p<0.05) after FDR correction was utilized as the strict threshold for definitive statistical significance. Crucially, this multiple testing correction protocol was maintained uniformly across all downstream structural analytical arms—including both intact-lipid multivariate models and deconstructed class-specific sub-molecular remodeling matrices—ensuring methodology-level consistency throughout the manuscript. The resulting differential data streams were visualized using volcano plots and multi-comparison scatter profiles.

### 2.14. Dynamic Trend Clustering Analysis

To delineate continuous abundance trajectories across the sequence of health, acute infarction, and post-PCI recurrence (HC→AMI→PRMI), the Fuzzy C-Means (FCM) clustering algorithm was deployed. Based on the mean standardized expression profile of each lipid entity, co-regulated molecules were partitioned into five dynamic trend modules. The clustering outputs were graphically expanded using a combination of line trajectory plots, localized heatmaps, and a bipartite main-class network layout.

### 2.15. Knowledge-Based Lipid–Protein–Pathway Contextualization

To translate abstract differential features and dynamic lipid modules into organized biological context, a knowledge-based mapping procedure was introduced. Validated lipid species were mapped to lipid-associated proteins using database-supported cross-references derived from SwissLipids, LIPID MAPS, UniProt, and related curated biochemical repositories. The compiled target protein sets were submitted to GO terms and KEGG pathway enrichment analyses utilizing the R package clusterProfiler. In strict alignment with exploratory guidelines, the resulting outputs were interpreted as computationally inferred, database-supported biological contexts (hypothesis prioritization) rather than direct experimental evidence of target binding, physical engagement, or pathognomonic pathway activation within this patient cohort. The standardized multi-stage evidence chain is documented in Supplementary Materials.

## 3. Results

### 3.1. Systemic Variations in the Serum Lipidomic Profile During Myocardial Infarction Remodeling

To characterize the lipidomic alterations associated with the occurrence and recurrence of myocardial infarction (MI), untargeted lipidomics was performed on serum samples from three cross-sectional cohorts including healthy controls (HC, *n* = 50), patients with acute myocardial infarction (AMI, *n* = 50), and patients with post-PCI recurrent myocardial infarction (PRMI, *n* = 35), whose baseline clinical and demographic characteristics are comprehensively summarized in Table 1. The evaluation of analytical platform stability and quality control (QC) performance is detailed in Supplementary Figure S1.

**Figure 2:**
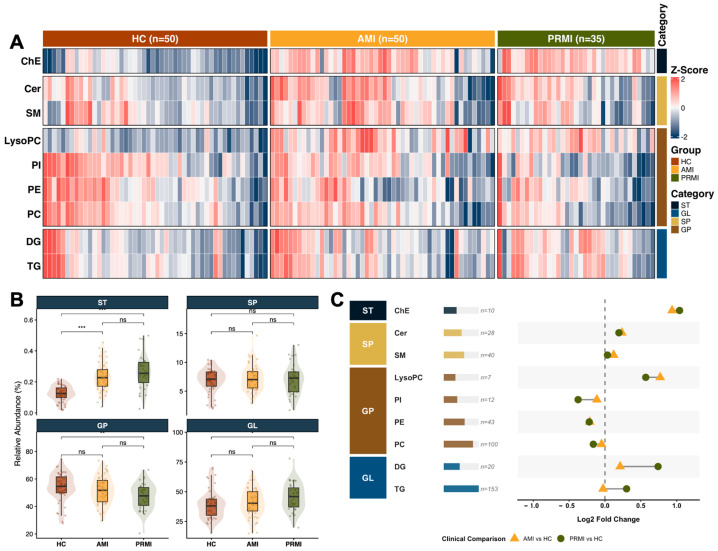
**Characterization of the serum lipidomic profiles across clinical groups during myocardial infarction remodeling.** (**A**) Heatmap representing the abundance profiles of nine major lipid subclasses across 135 serum samples (50 HC, 50 AMI, and 35 PRMI). Each column represents an individual sample, and each row represents a lipid subclass grouped by its respective lipid category. Color intensity corresponds to the z-score normalized values, with blue indicating lower abundance and red indicating higher abundance. (**B**) Comparison of the relative abundance percentages of the four major lipid categories across the three clinical groups. Combined violin and box plots illustrate the distribution of relative percentage content for sterol lipids (ST), sphingolipids (SP), glycerophospholipids (GP), and glycerolipids (GL). Each point represents an individual biological sample. Statistical significance between groups was determined using the two-tailed Wilcoxon rank-sum test. Significance levels (Benjamini–Hochberg FDR-adjusted): ns, *p* > 0.05; *, *p* ≤ 0.05; **, *p* ≤ 0.01; ***, *p* ≤ 0.001. (**C**) Subclass-level molecular identification counts and fold-change distributions relative to the healthy control group. Left: horizontal bar plot indicating the number of unique lipid species identified within each subclass, with colors matching their parent lipid categories. Right: plot illustrating the mean log2 fold changes for the AMI group (yellow triangles) and the PRMI group (green circles) relative to the HC group, with error bars representing the standard error of the mean. The vertical dashed line at log2 fold change = 0 indicates no change.

At the subclass level, the nine major lipid subclasses identified across the cohorts exhibited coordinated variations in their relative peak area profiles (Figure 2A). The individual-level global expression profiles are displayed in Supplementary Figure S2E.

To assess compositional shifts at the category level, we evaluated the relative signal contribution of the four major lipid categories, calculated as the percentage of the summed normalized peak areas for each category (Figure 2B). Compared with the HC group, glycerolipids (GL) and sterol lipids (ST) exhibited increasing trends in their relative signal proportions in both the AMI and PRMI groups. Specifically, the relative peak area proportion of ST increased from a low baseline in the HC group to higher levels in the disease groups (*p* ≤ 0.001). Conversely, glycerophospholipids (GP) showed a significant relative decrease in signal proportion in the disease groups (*p* ≤ 0.01). These trends indicate a shift in the relative lipidomic signal from membrane associated lipids toward storage and signaling lipids during myocardial infarction remodeling.

*p* values were calculated using Fisher’s exact test or the chi-square test for sex distribution, as appropriate, and one-way ANOVA for chronological age. Pairwise comparisons are provided in Supplementary Table S1. Statistical Power Note: Due to the retrospective nature of this exploratory clinical cohort, a formal a priori sample size calculation was not executed. However, secondary post hoc power analysis via the R pwr package demonstrated that the total sample volume holds an exceptionally robust statistical power of 98.92% for global cross-group ANOVA variations (at a large effect size f = 0.40, α = 0.05) and 94.82% for the localized PRMI (*n* = 35) vs. AMI (*n* = 50) pairwise contrasts (at Cohen’s d = 0.80). Conversely, the sex-stratified subgroup baseline contrast (*n* = 2 in female HC vs. *n* = 10 in female PRMI) is severely under-powered (15.49% at d = 0.80), and thus all female-disaggregated reporting trends are explicitly designated for descriptive within-cohort exploration only, rather than generalizable clinical biomarker claims.

We next evaluated these variations at the molecular subclass level by analyzing the number of identified lipid species and their relative fold changes based on normalized peak areas (Figure 2C). A total of 413 lipid species were monitored, including 153 triglycerides (TG), 100 phosphatidylcholines (PC), and 43 phosphatidylethanolamines (PE). Consistent with the category-level profiles, cholesteryl esters (ChE, *n* = 10) within the ST category, along with triglycerides (TG) and diacylglycerols (DG, *n* = 20) within the GL category, showed positive fold changes in peak areas (log2fold change > 0) in both the AMI and PRMI groups relative to the HC group. In contrast, major membrane lipid subclasses within the GP category, such as PC, PE, and phosphatidylinositols (PI), consistently exhibited negative fold changes (log2fold change < 0). Notably, the relative fold changes in TG, DG, and PI were more pronounced in the PRMI group than in the AMI group, suggesting distinct or persistent lipid alterations in patients experiencing recurrent events.

### 3.2. Category-Specific LDA for Prioritizing Discriminatory Lipid Features

**Figure 3:**
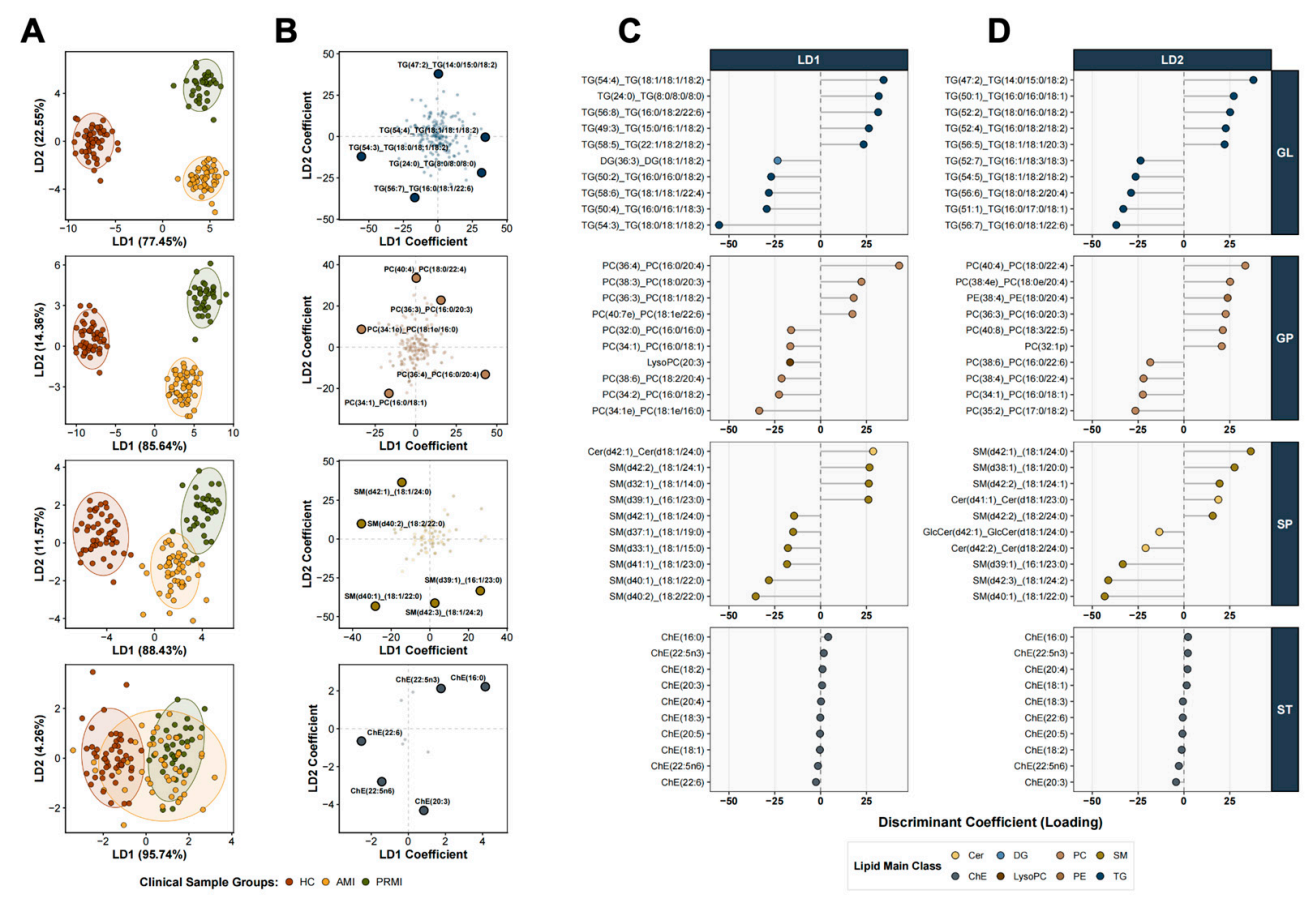
**Category-specific Linear Discriminant Analysis projections and prioritized lipid contributors across major lipid categories.** (**A**) Exploratory LDA scores plots representing sample projections onto the first two discriminant axes for glycerolipids, glycerophospholipids, sphingolipids, and sterol lipids. Ellipses represent the ninety-five percent confidence intervals for healthy controls, patients with acute myocardial infarction, and patients with post-PCI recurrent myocardial infarction. Individual data points represent biological replicates colored by clinical groups. (**B**) Loading spaces of the corresponding LDA models. Grey background circles represent all identified lipid species within each category, while large colored circles highlight the top five key contributing lipid molecules prioritized by their multidimensional Euclidean coordinate distance from the origin. (**C**,**D**) Standardized discriminant coefficients for the top ten highest-contributing lipid molecules on the LD1 (**C**) and LD2 (**D**) axes, respectively. The segment lengths and points represent the magnitude and direction of the loading coefficients. Points are colored by lipid main class.

Following the global analysis that revealed overall trends, the first major branch of our analytical paradigm—Intact Lipid-Level Analysis—aims to characterize intact lipid molecules from three complementary analytical perspectives. First, to evaluate within-cohort lipid remodeling patterns, we employed category-specific Linear Discriminant Analysis as an exploratory dimension-reduction tool to prioritize lipid features contributing to group separation from the complex pool of lipid molecules. To this end, we employed a category-specific Linear Discriminant Analysis (LDA) approach, with the main category-specific LDA outputs summarized in Figure 3 and supporting robustness analyses provided in Supplementary Figures S3–S6. This approach does not perform a single global analysis on all lipids but instead constructs an independent discriminant model for each major lipid category, thereby enabling a more refined characterization of category-specific lipidomic patterns associated with the clinical groups.

Figure 3 summarizes the LDA results for four major lipid categories: ST, SP, GP, and GL. The central scatter plot in each sub-figure shows the projection of samples onto the two main discriminant axes (LD1 and LD2). These category-specific models showed within-cohort separation patterns among the study groups and helped prioritize the lipid features contributing most strongly to the discriminant axes. A consistent pattern across all models was that the LD1 axis primarily captured the differences between HC and the disease states (AMI/PRMI), while the LD2 axis mainly distinguished between the two distinct disease stages, acute MI (AMI) and PRMI.

The peripheral bar plots (Figure 3C,D) quantify and display the lipid molecules contributing most strongly to the discriminant axes, based on the magnitude of their LDA coefficients. This feature supports LipiDecipher’s difference-attribution step by highlighting lipid molecules that contribute strongly to the observed within-cohort group contrasts. In the ST category (Figure 3A), although there was slightly more overlap between groups, the model (LD1 contribution 95.74%) still showed partial separation between the HC and PRMI groups, driven primarily by the downregulation of various ChE, such as ChE (20:5) and ChE (22:6). In the SP category (Figure 3A), the distinction between “health vs. disease” (LD1 contribution 88.43%) relied mainly on various long-chain SM and ceramides (Cer). Specifically, molecules such as SM (d42:1) and Cer (d41:1) contributed prominently to the separation of AMI/PRMI samples from HC samples. In the GP category (Figure 3A), the major contributors to the disease-versus-control discriminant axis (primarily reflected by the LD1 axis, 85.64% contribution) were mainly the downregulation of various polyunsaturated fatty acid (PUFA)-containing phospholipids, such as PC (38:6), PC (38:4), and PI (34:1). In the GL category (Figure 3A), the GL model was driven predominantly by various TG and diacylglycerol (DG) species. For instance, TG (54:3) and DG (36:3) were significantly elevated in the disease groups, representing major contributors to the disease-versus-control discriminant axis.

The category-specific LDA provided an internal, category-wise view of group separation and prioritized lipid molecules with strong contributions to the discriminant axes within this cohort. Sample-level LDA scores, full LDA coefficients, and the top contributors displayed in Figure 3 are provided in Supplementary Tables S6 and S7. To evaluate robustness, we further performed resampling-based feature stability assessment, repeated stratified 5-fold cross-validation, and permutation testing (Supplementary Figures S3–S5). Apparent separation metrics are provided for descriptive visualization of within-cohort clusters and should not be interpreted as generalizable clinical classification performance. Together, these analyses indicate that the category-specific projections capture stable, reproducible differences in lipid profiles within this study population, while independent cohort validation remains necessary before clinical utility can be proposed (Supplementary Figure S6). Together, these analyses indicate that the category-specific lipid profiles contain internally reproducible discriminatory information beyond random label structure, while independent cohort validation remains necessary before clinical classifier claims can be made.

### 3.3. Differential Comparison and Functional Mapping: Quantifying Changes at Critical Stages

**Figure 4:**
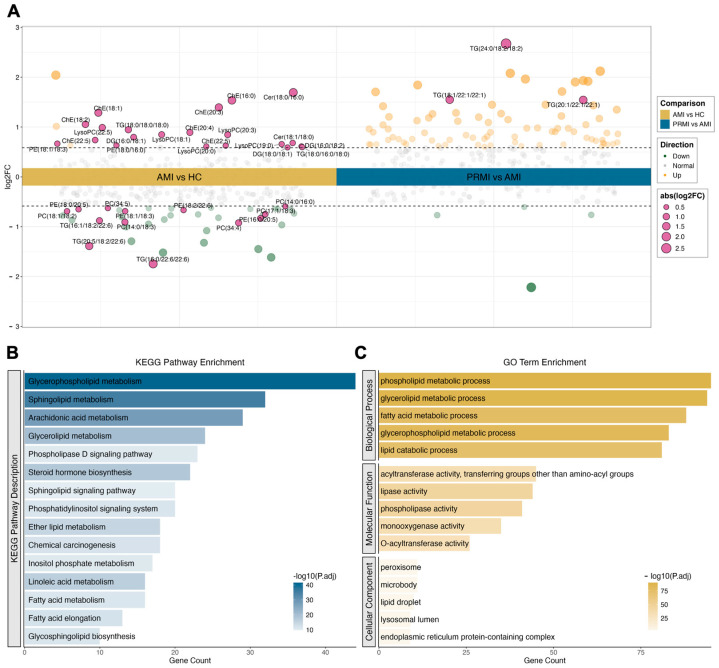
Differential lipid signatures and database-supported biological contextualization in myocardial infarction recurrence. (**A**) Multi-comparison overview plot juxtaposing two key comparisons: AMI vs. HC and PRMI vs. AMI. Point color indicates the direction of change, and point size is proportional to the absolute log2FC. Lipids meeting the significance criteria are highlighted and labeled. (**B**) KEGG pathway enrichment analysis based on database-mapped lipid-associated protein sets derived from differential lipids identified between PRMI and AMI groups. Bar length represents the number of mapped proteins and color intensity corresponds to enrichment significance. (**C**) Gene Ontology (GO) enrichment analysis, faceted by Biological Process, Cellular Component, and Molecular Function. These enrichment results represent putative biological contexts and should not be interpreted as direct evidence of lipid–protein binding, target engagement, or pathway activation.

After LDA provided an overview of key discriminatory features, we next examined lipid changes from a pairwise-comparison perspective. We used statistical testing to quantify lipids that changed significantly during two critical transitions: disease onset (AMI vs. HC) and disease progression/recurrence (PRMI vs. AMI) (Figure 4A). Identifying differential lipids, however, is only the first step. To place these lipids into a more interpretable biological context, we incorporated a knowledge-based lipid–protein–pathway mapping procedure.

Using the differential lipids identified in the “PRMI vs. AMI” comparison as input, we mapped these lipids to curated lipid-associated proteins using database-supported lipid annotations and cross-references from SwissLipids and LIPIDMAPS-related resources. These database-mapped lipid-associated protein sets were then used for downstream enrichment analysis with clusterProfiler. The complete evidence chain linking differential lipids to mapped lipid-associated proteins and enriched KEGG/GO annotations, including database identifiers, mapping sources, evidence types, PMID availability where present, and validation status in the present study, is provided in Supplementary Materials.

The resulting enrichment outputs prioritized several biologically plausible contexts associated with lipid differences between PRMI and AMI. KEGG analysis (Figure 4B) prioritized pathways including glycerophospholipid metabolism, sphingolipid metabolism, and arachidonic acid metabolism. GO analysis (Figure 4C) further identified annotations related to phospholipid metabolism, fatty acid metabolism, acyltransferase-related functions, peroxisomes, and lipid droplets. These results are compatible with altered membrane remodeling, inflammatory signaling, and energy-related processes, but they should be interpreted as hypothesis-prioritizing annotations rather than as direct validation of lipid–protein–pathway relationships in the present cohort.

In summary, this analysis step identified recurrence-associated differential lipids and organized them into candidate biological contexts through database-supported lipid–protein–pathway mapping. Its value lies in biological contextualization and hypothesis prioritization, while experimental follow-up will be required to determine whether these inferred associations reflect direct functional mechanisms in myocardial infarction recurrence.

### 3.4. Dynamic Trend Clustering: Delineating Co-Regulated Lipid Modules

Although pairwise differential analysis can pinpoint metabolic variations at specific transitions, it cannot fully capture the continuous trajectories of lipid features across the complete cross-sectional spectrum. Therefore, as a complementary dynamic analytical layer, we employed the Fuzzy C-Means (FCM) clustering algorithm to segregate co-regulated lipid modules across the clinical spectrum (HC→AMI→PRMI).

**Figure 5:**
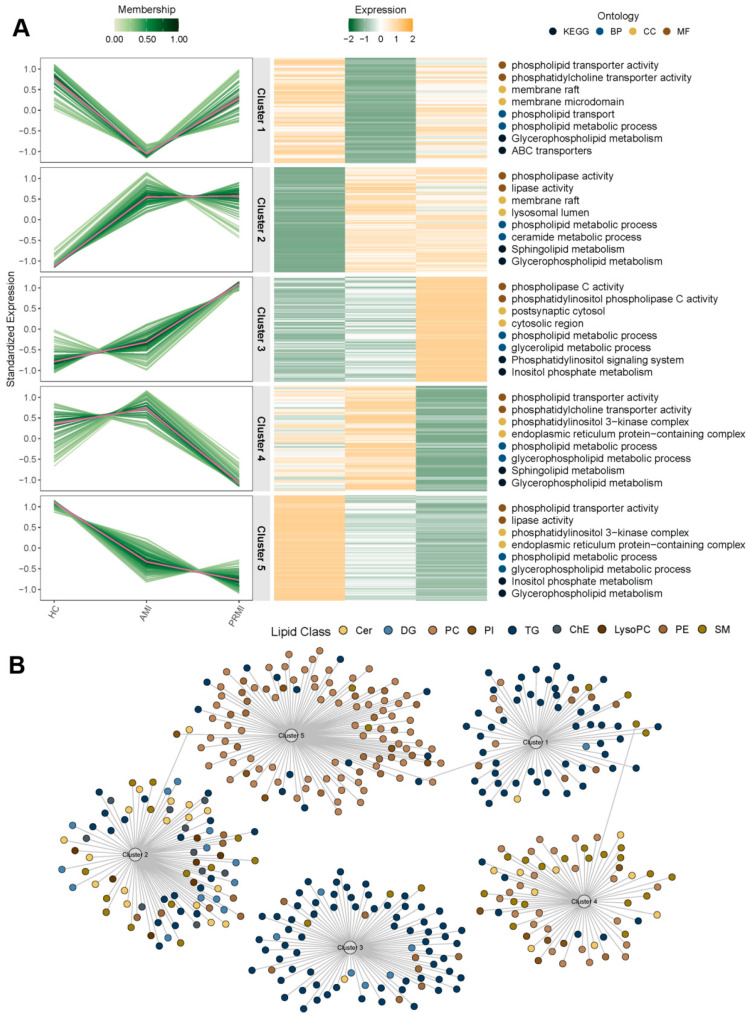
**Dynamic clustering reveals co-regulated lipid modules and database-supported enrichment annotations during myocardial infarction progression.** (**A**) Fuzzy C-Means (FCM) clustering of lipid abundance trajectories across HC, AMI, and PRMI groups. Left panels show standardized expression trajectories and cluster-level heatmaps. Right panel shows enrichment annotations based on database-mapped lipid-associated protein sets from lipids within each cluster. Each dot represents an enriched KEGG pathway or GO term, and dot color indicates the ontology source. These annotations are intended for hypothesis generation and do not indicate experimentally validated pathway activation or inhibition. (**B**) Bipartite network connecting lipid species to their assigned expression clusters. Node colors indicate lipid main classes.

This mathematical clustering partitioned the modulated lipidome into five distinct modules characterized by divergent kinetic patterns, which are organized chronologically into a sequential biological narrative in Table 2. As detailed in Figure 5A (left panels), each cluster outlines a distinct disease-associated behavior: (1) Cluster 3 (Sustained/Progressive Upregulation) captures lipids that elevate acutely during onset (AMI) and compound further during recurrence (PRMI), dominated primarily by storage-related triacylglycerols (TG) and specific phosphatidylcholines (PC). (2) Cluster 2 (Infarction-Specific/Transient Peak) highlights an acute metabolic surge unique to the window of myocardial injury (AMI) which successfully returns toward baseline in the recurrent stage (PRMI), driven chiefly by diacylglycerol (DG) intermediates. (3) Cluster 1 (V-shaped/Transient Decline) delineates an initial drop during acute injury followed by an adaptive or compensatory recovery in the post-PCI phase. (4) Cluster 4 (Gradual/Mild Downward) reflects a continuous but milder structural attrition across the disease timeline. (5) Cluster 5 (Sustained/Progressive Downregulation) isolates lipid features that suffer permanent suppression immediately following the primary ischemic event and fail to recover during recurrence, populated prominently by signaling ceramides (Cer) and selected TG fractions. Through this chronologically mapped framework, individual lipids are grouped into co-regulated functional blocks, facilitating more interpretable biological contextualization.

These dynamic modules provided a basis for organizing lipids with similar abundance trajectories into database-supported biological contexts through the lipid–protein–pathway mapping strategy. By performing independent pathway enrichment analysis on each cluster module (Figure 5A, right panel), we found that lipids with different dynamic patterns were associated with distinct putative biological annotations: For example, the mapped lipid-associated proteins linked to lipids in the sustainedly upregulated Cluster 3 were enriched for annotations such as the phosphatidylinositol signaling system and glycerolipid metabolic processes, suggesting candidate contexts related to signaling and metabolic remodeling. By contrast, lipids in the sustainedly downregulated Cluster 5 were associated with lipase activity and the phosphatidylinositol 3-kinase complex, suggesting candidate associations with lipid hydrolysis and phosphoinositide-related signaling contexts.

To further summarize the molecular composition of these dynamically defined modules, we constructed a bipartite network graph connecting lipids to their clusters (Figure 5B). This network displays the lipid main-class composition within each dynamic module. For instance, the network shows that the sustainedly upregulated Cluster 3 is primarily composed of TG and PC, while the sustainedly downregulated Cluster 5 is enriched in Cer and TG. This analysis identified co-regulated groups of lipids and provided an integrated view of their dynamic patterns, chemical classes, and database-supported biological annotations.

### 3.5. Structurally Resolved Analysis of Deconstructed Fatty-Acyl Chains and Remodeling Indices

To evaluate sub-molecular structural variations beyond intact lipid profiles, parent lipid relative abundances were deconstructed into their constituent fatty-acyl chains. This deconstruction proportionally attributes parent lipid normalized peak areas to individual chains based on molecular weight. To minimize potential false-positive annotations from spectral noise, deconstructed chains with carbon lengths greater than 27 were excluded from this analysis. Because these deconstructed summaries are derived from complex lipid pools (e.g., triglycerides, glycerophospholipids, and sphingolipids), they are defined as relative remodeling indices rather than direct measurements of circulating free species.

**Figure 6:**
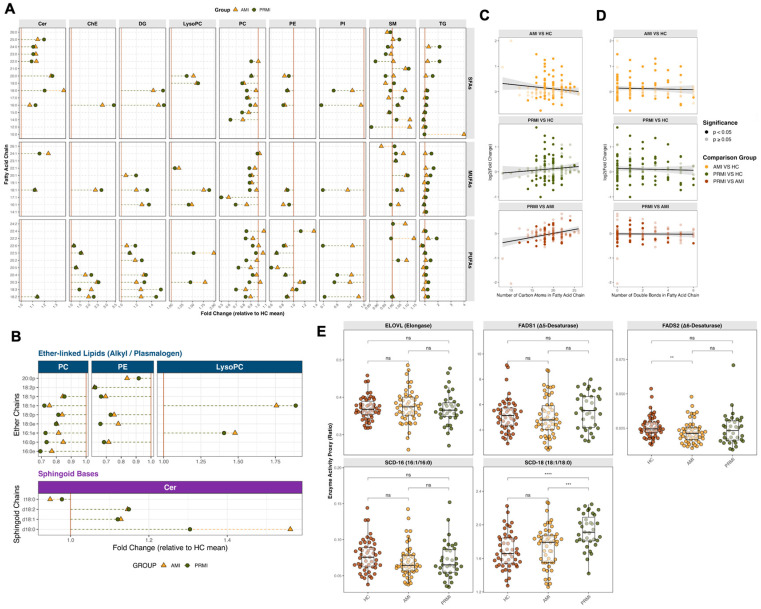
**Structurally resolved deconstruction of fatty acyl chains, structure–abundance correlations, and fatty-acyl remodeling proxies across clinical groups.** (**A**) Standardized relative fold changes in individual standard fatty acyl chains relative to the healthy control (HC) group across nine major lipid classes. Individual segments and points represent deconstructed chains (Saturated Fatty Acids, SFAs; Monounsaturated Fatty Acids, MUFAs; Polyunsaturated Fatty Acids, PUFAs) partitioned from parent lipid normalized peak areas. Triangles represent AMI vs. HC and circles represent PRMI vs. HC. (**B**) Relative abundance variations in deconstructed special structural modifications. Upper panel: deconstructed ether-linked chains (alkyl/plasmalogen, designated with e or p) within PC, PE, and LysoPC. Lower panel: deconstructed sphingoid bases (designated with d or t) within Ceramides (Cer). Points indicate the mean fold changes relative to the HC group. (**C**) Correlation analysis between the deconstructed fatty-acyl carbon chain length (number of carbon atoms) and relative log2 fold changes across three pairwise group comparisons (AMI vs. HC, PRMI vs. HC, and PRMI vs. AMI). Linear regression fits are indicated by the solid lines, with shaded areas representing the 95% confidence intervals. Points are colored by significance (*p* < 0.05 in black; *p* ≥ 0.05 in grey). (**D**) Correlation analysis between the deconstructed fatty-acyl double bond count and relative log2 fold changes across the clinical comparisons. Solid lines represent the linear regression fits with 95% confidence intervals. (**E**) Comparison of estimated fatty-acyl remodeling ratios (product-to-substrate proxies) for ELOVL (18:0/16:0), FADS1 (20:4/20:3), FADS2 (18:3/18:2), SCD-16 (16:1/16:0), and SCD-18 (18:1/18:0) across the HC, AMI, and PRMI groups. Individual points represent single biological replicates. Box plots represent median and interquartile ranges, while violin plots show density distributions. Group differences were evaluated using the Wilcoxon rank-sum test. Significance levels (Benjamini–Hochberg FDR-adjusted): ns, *p* > 0.05; **, *p* ≤ 0.01; ***, *p* ≤ 0.001; ****, *p* ≤ 0.0001.

Standardized relative abundance profiles of these deconstructed chains across all samples are visualized in a global heatmap (Supplementary Figure S7). To examine these variations in detail, relative fold changes in individual deconstructed chains within major lipid classes were evaluated across the clinical groups (Figure 6A,B). Analysis of standard acyl chains revealed complex, backbone-dependent profiles (Figure 6A). Within the glycerophospholipid (GP) family, marked heterogeneity was observed. While most deconstructed fatty acids within phosphatidylcholines (PC) and phosphatidylethanolamines (PE) remained stable or decreased in the disease groups, those in lysophosphatidylcholines (LysoPC) showed general relative increases. Notably, phosphatidylinositols (PI) demonstrated progressive, disease-stage-associated relative decreases in several polyunsaturated fatty acid (PUFA) chains, with more pronounced reductions in the PRMI group than in the AMI group. Within glycerolipids, triglycerides (TG) and diacylglycerols (DG) displayed relative increases across most standard acyl chains. However, specific signatures such as TG-associated 10:0 showed a transient relative increase in the AMI group but returned toward baseline levels in the PRMI group. Fatty-acyl chains in Ceramides (Cer) and cholesteryl esters (ChE) also exhibited general relative increases, particularly within their polyunsaturated fractions, whereas sphingomyelins (SM) showed highly chain-specific patterns. Parallel evaluation of special structural modifications, including ether-linked chains and sphingoid bases, highlighted distinct pathological responses (Figure 6B). In ether-linked PC, PE, and LysoPC subclasses, deconstructed ether-linked chains (designated with e or p suffixes) exhibited subclass-specific variations. Within the Cer subclass, deconstructed sphingoid bases (such as d18:0 and d18:1) consistently exhibited higher relative abundance levels in both the AMI and PRMI groups relative to the healthy controls.

To evaluate global structural trends, structure–abundance correlation analyses were performed between fatty acyl structural parameters (carbon chain length and double-bond count) and relative log2fold changes. In the AMI vs. HC and PRMI vs. HC comparisons, carbon chain length displayed a positive linear correlation with relative fold changes (Figure 6C), reflecting a relative shift toward longer fatty-acyl chains during myocardial events. Conversely, double-bond count did not show a consistent global correlation across clinical transitions (Figure 6D), suggesting that unsaturation-related variations are highly heterogeneous and class-dependent.

To complement these global structural assessments, product-to-substrate ratios were calculated as proxies for fatty acid remodeling pathways (Figure 6E). Compared with the HC group, the SCD-18 proxy (18:1/18:0 ratio) was significantly elevated in both the AMI and PRMI groups (Adj. *p* < 0.001). In contrast, the estimated remodeling ratios for elongase (ELOVL, 18:0/16:0), FADS1 (20:4/20:3), and SCD-16 (16:1/16:0) did not exhibit statistically significant differences after Benjamini–Hochberg FDR correction across the clinical groups, while FADS2 (18:3/18:2) displayed a modest alteration in the AMI group (*p* < 0.01). These patterns suggest that alterations in desaturation-related product-to-substrate balances, as represented by the SCD-18 proxy, participate in the systemic lipid remodeling associated with myocardial injury, although direct enzymatic assays would be required for functional validation.

In summary, the integration of structural deconstruction, class-specific analysis, structure–abundance correlations, and remodeling proxies characterizes myocardial-infarction-associated relative lipid changes across multiple structural dimensions. These coordinated shifts in fatty-acyl backbones, chain lengths, and inferred biosynthetic ratios provide a detailed, structured view of the circulating lipidome in this clinical cohort, offering hypothesis-generating signatures for subsequent functional validation.

## 4. Discussion

In this study, we applied the LipiDecipher analytical framework to a retrospective lipidomics dataset of myocardial infarction. This case study illustrates how the framework can organize complex lipidomics data into multiple interpretable analytical levels, prioritize candidate lipid signatures, and generate biologically plausible, database-supported hypotheses. More broadly, it provides a practical workflow for moving beyond lists of differential lipids toward more structured interpretation of lipidomic variation.

LipiDecipher: A Synergistic Strategy Integrating Multi-Source Information and Deconstructing Lipid Structure.

Accurate lipid identification and annotation serve as the critical link between raw data and biological meaning in lipidomics research. However, the inherent limitations of single databases and the structural complexity of lipids often lead to incomplete or inaccurate annotations [12,13]. LipiDecipher addresses these challenges through two core designs. In contrast to existing functional lipidomics tools (such as LION-web or Lipid Mini-On) that primarily focus on standard pathway enrichment or descriptive taxonomic grouping based on categorical intact-lipid lists [14,15], LipiDecipher bridges statistical prioritization with continuous sub-molecular architectural dissection.

Firstly, LipiDecipher integrates multiple authoritative databases [16,17,18], including LIPIDMAPS and SwissLipids, to improve annotation coverage and provide complementary identifiers for downstream analyses. By reducing reliance on a single database, this approach helps mitigate the “information silo” problem arising from cross-database discrepancies and provides a structured annotation basis for subsequent lipid–protein–pathway contextualization.

Second, a central feature of LipiDecipher is its lipid deconstruction capability. Whereas conventional analyses often stop at intact lipid species such as TG (54:3), this framework further summarizes those signals into constituent fatty acyl-chain features. It is highly noteworthy that this computational projection possesses a unique biological advantage over wet-lab chemical hydrolysis coupled with gas chromatography–mass spectrometry. While chemical hydrolysis measures the absolute physical pool of fatty acids, it completely forfeits the biological class of origin, rendering it impossible to distinguish whether an acyl chain such as palmitate was derived from membrane-bound phospholipids or storage-bound neutral lipids. LipiDecipher’s virtual remodeling indices effectively address this physical limitation by computationally dissecting the esterified lipid pool while preserving the structural metadata of the parent headgroup. As illustrated in Figure 6, this enables lipidomic variation to be examined across interpretable structural levels, including class-specific fatty acyl composition, chain length, and degree of unsaturation. Rather than directly revealing mechanism, this additional analytical layer helps expose structure-related patterns that may inform downstream biological interpretation and hypothesis prioritization.

### 4.1. Systematic Lipid Remodeling: From Global Landscape to Dynamic Modules

Our investigation began at a global level, showing broad lipidomic remodeling during MI and its recurrence. A salient feature was the fundamental shift in lipid composition from healthy controls to AMI and PRMI patients: a progressive increase in the relative abundance of glycerolipids (GL) for energy storage, with a concomitant decrease in glycerophospholipids (GP), the structural backbone of cell membranes (Figure 2B). This observation suggested a recurring theme in our dataset: MI was associated with an altered balance between glycerolipid-rich storage-related signals and glycerophospholipid-rich membrane-associated signals.

To comprehensively capture the key molecules driving this shift, we employed a multi-dimensional screening strategy. Category-specific LDA (Figure 3) prioritized lipid features contributing strongly to within-cohort group separation. For example, the downregulation of PUFA-containing phospholipids (e.g., PC (38:6), PI (34:1)) and the upregulation of long-chain sphingolipids (e.g., SM (d42:1), Cer (d41:1)) emerged as major contributors to the disease-versus-control discriminant axis. Dynamic clustering (FCM, Figure 5) further revealed co-regulated lipid modules. For example, the sustainedly upregulated Cluster 3, primarily composed of TG and PC species, was associated through database-supported mapping with annotations such as the phosphatidylinositol signaling system and glycerolipid metabolic processes. Conversely, the sustainedly downregulated Cluster 5, enriched in Cer and TG species, was linked to annotations related to lipase activity. These observations suggest candidate biological contexts for follow-up studies, but they do not establish direct pathway activation or inhibition.

Taken together, this progressive analysis consistently highlighted lipid classes such as TG, PC, and Cer as important contributors to MI-associated lipid remodeling in this cohort, providing candidates for subsequent mechanistic exploration.

### 4.2. “Lipid–Protein–Pathway” Mapping: Database-Supported Biological Contextualization in Lipidomics

One of the major bottlenecks in current lipidomics research is linking identified differential lipids to specific biological pathways [19,20]. To address this challenge, we incorporated a knowledge-based lipid–protein–pathway contextualization strategy. Differential lipids were mapped to lipid-associated proteins using curated database resources such as SwissLipids [18] and LIPIDMAPS-related annotations, enabling established enrichment tools to be used for organizing lipid signatures into putative biological contexts.

In our study (Figure 4), we applied this strategy to focus on the differential lipids most closely associated with MI recurrence (PRMI vs. AMI). The corresponding database-mapped lipid-associated protein sets were enriched for pathways including Glycerophospholipid metabolism, Sphingolipid metabolism, and Arachidonic acid metabolism. These findings are compatible with prior literature implicating glycerophospholipid remodeling in cardiomyocyte injury and dysfunction [21], where phospholipase A2 (PLA2) families play a core role. Specifically, cytosolic phospholipase A2 (cPLA2) regulates arachidonic acid and lysophosphatidylcholine (LPC)-derived signaling pathways, magnifying inflammatory lipid mediators and accelerating vascular injury [22]. Concurrently, secretory phospholipase A2 (sPLA2) overexpresses during inflammation, hydrolyzing the sn-2 position of phospholipids to generate LPC and free fatty acids that mediate cascade reactions [23]. Lipoprotein-associated phospholipase A2 (Lp-PLA2/PLA2G7) also acts as an immunomodulatory hub, with its activity promoting macrophage migration and cardiac inflammation-associated remodeling [24,25]. Imbalances in membrane phospholipid metabolism are further supported by literature showing elevated LPC accumulation alongside decreased phosphatidylcholines in injured myocardium [26].

Furthermore, our mapping prioritized sphingolipid metabolism—especially ceramide-related pathways—which are heavily implicated in apoptosis and inflammation [27,28]. This aligns with prior literature establishing core bioactive sphingolipids, particularly those containing a d18:1 sphingoid base backbone (such as Cer d18:1/16:0, Cer d18:1/24:1, and Cer d18:1/24:0), as prominent clinical biomarkers for adverse prognoses in cardiovascular patients [29]. Mechanistically, ceramide accumulation triggers mitochondrial dysfunction, activates caspase-dependent apoptotic pathways, and exacerbates cardiac lipotoxicity and subsequent fibrosis during post-MI ventricular remodeling [30,31,32]. These deleterious cascades are interconnected with upstream enzymatic regulators, as targeting the serine palmitoyltransferase long chain base subunit 2 (SPTLC2) to block de novo ceramide synthesis has been shown to specifically alleviate cardiomyocyte apoptosis and preserve cardiac function [32]. This accumulated lipotoxicity directly drives sterile inflammation—characterized by native immune activation, macrophage infiltration, and tissue fibrosis—disrupting organelle configurations like mitochondrial-associated membranes (MAM) and serving as a key pathological link bridging acute syndromes to adverse myocardial remodeling [33,34,35,36,37]. Additionally, GO enrichment analysis identified annotations related to lipid droplets and peroxisomes, which are biologically consistent with the observed TG-associated remodeling and with pathways regulating lipid storage and oxidative metabolism [34,38,39,40,41,42].

### 4.3. Structural Deconstruction: Generating Structure-Level Hypotheses from Fatty Acyl Chain Remodeling

Through LipiDecipher’s deconstruction analysis, we extended the analysis of intact lipids to examine associations between fatty acyl-chain structure and disease state. Because these fatty acyl summaries were derived from complex lipid pools rather than measured as free fatty acids, they should be interpreted as structure-weighted representations of lipid remodeling. This analysis revealed several class-dependent and structure-associated patterns. First, the structure–abundance correlation analysis showed that the carbon chain length of fatty acyl chains was positively correlated with abundance changes (Figure 6C), reflecting a relative shift toward longer-chain fatty acyl species during MI-associated remodeling. Conversely, the double-bond count did not exhibit a consistent global correlation across clinical transitions (Figure 6D), suggesting that unsaturation-related variations are highly heterogeneous and strictly class-dependent. However, these global correlations do not directly measure elongase or desaturase activities.

To investigate this complexity, we calculated fatty-acyl remodeling ratios as enzyme-related proxies in Figure 6E. The results showed that the SCD-18 product-to-substrate proxy was significantly elevated, whereas other desaturase-related proxies did not differ significantly, which aligns with existing literature on the role of SCD1 in cardiovascular protection and anti-inflammation [43,44]. In baseline biochemistry, stearoyl-CoA desaturase 1 (SCD1) is the rate-limiting enzyme that converts saturated fatty acids (SFAs, e.g., 18:0) into monounsaturated fatty acids (MUFAs, e.g., 18:1) [45]. Prior non-cardiovascular literature indicates that SCD1-mediated desaturation remodeling enhances lipid membrane fluidity, mitigates ferroptosis, suppresses lipid peroxidation, and preserves endoplasmic reticulum integrity and mitochondrial respiration, thereby counteracting SFA-induced cellular lipotoxicity and injury cascades [46,47].

Crucially, however, we must maintain extreme epistemic caution regarding this proxy readout. The current mechanistic evidence demonstrating that SCD1 activity directly counteracts lipotoxicity or endoplasmic reticulum stress originates almost exclusively from oncology and general metabolic disease models; there is currently no direct experimental or causal evidence establishing that circulating desaturation indices exert a definitive cardioprotective effect or dictate clinical outcomes in human coronary syndromes. Furthermore, circulating fatty acid ratios function as a systemic metabolic sink, continuously modulated by extra-cardiac factors including de novo hepatic lipogenesis, adipose tissue lipolytic mobilization, and clinical baseline characteristics (such as age, sex, and fasting states, which can account for substantial lipidomic variance with partial-r up to 0.19) [48,49,50]. Background pharmacological interventions may also act as potential confounders; although aggressive lipid-lowering trials (e.g., PCSK9 inhibitors) have demonstrated capacity to modulate patient outcomes, there is currently no direct evidence delineating how baseline statin therapies interact with or confound specific circulating oleic-to-stearic acid ratios or native SCD1 expressions [51,52].

Notably, the class-specific deconstruction analysis (Figure 6A,B) revealed fine-grained remodeling patterns that may be missed by intact-lipid analysis alone:

Candidate recurrence-associated PI remodeling pattern: PI-associated fatty acyl chains showed a progressive decrease from AMI to PRMI, suggesting a candidate recurrence-associated remodeling pattern within the PI lipid pool. The biological basis for this inference lies in the central role of PI and its phosphorylated derivatives as key nodes in cellular signaling networks. For instance, the critical signaling molecule PtdIns(3,4,5)P_3_ regulates cell survival, proliferation, and response to mechanical stress by activating downstream proteins—processes crucial for cardiomyocyte repair after ischemic injury [53,54]. Literature corroborates that the selective loss or structural rearrangement of long-chain PUFAs in membrane phospholipids (such as PC, PE, and PI) alters the physical properties of the lipid bilayer, precipitating increased membrane rigidity and disrupting lipid raft micro-domains [55,56,57]. Accumulating literature on structure–function relationships demonstrates that phospholipid acyl chain unsaturation and double-bond positions actively modulate membrane packing heterogeneity, where specific polyunsaturated PE species promote submicron domain segregation and guide membrane fluidity under high metabolic demands [58,59]. This biophysical collapse can secondarily perturb localized ion channel transport, calcium handling, and cell-to-cell communication, directly amplifying intracellular calcium overload and electrical instability in the ischemic myocardium [60,61,62,63]. This micro-environmental fragility is highly chain-specific; lipidomic evidence indicates that membrane PC and PE molecules containing highly unsaturated acyl chains (such as 18:2 or 20:4) exhibit pronounced susceptibility to reactive oxygen species, undergoing selective oxidative loss and releasing volatile aldehydes during severe tissue injury and chronic remodeling cascades [64,65,66]. Under severe pathological stress, a persistent depletion of PIP3 disrupts the essential PI3K-Akt survival signaling axis, bridging chronic pathway dysfunction to progressive myocardial fibrosis and cardiac dysfunction [67,68]. This observation provides a structure-level hypothesis linking PI pool remodeling to disease progression. However, this inference remains indirect and requires targeted phosphoinositide profiling and functional validation.

Candidate acute-phase TG-associated 10:0 pattern: TG-associated 10:0 showed a marked increase in the AMI group and returned toward control levels in PRMI, suggesting a candidate acute-phase remodeling pattern. This kinetic behavior is highly compatible with the bioinformatic behavior of Cluster 2 (Infarction-Specific Transient Peak) from our dynamic FCM modular discovery. From a metabolic standpoint, myocardial ischemia–reperfusion injury triggers severe mitochondrial dysfunction and lower expression of genes governing oxidative pathways, which severely impairs long-chain fatty acid β-oxidation [69]. Crucially, myocardial tissues natively lack medium-chain acyl-CoA synthetase (ACSM), which strictly limits the heart’s capacity to utilize MCFAs (C8–C12) via alternative β-oxidation pathways despite mitochondrial baseline respiratory affinities [70]. Under acute ischemic stress, these un-oxidized MCFAs are sequestered and temporarily “packaged” into triacylglycerols, provoking a transient, protective intracellular lipid accumulation unique to the acute infarction window [71]. As patients transition to the chronic post-PCI recurrence phase (PRMI), this acute adaptive response subsides, giving way to sustained neutral lipid overload and chronic accumulation. Large-scale longitudinal cohort data encompassing 51,719 patients confirm that cumulative exposure, absolute levels, and longitudinal variability of triglycerides are causally and independently associated with major adverse cardiovascular events (MACE), acting as a powerful independent risk factor driving coronary recurrence [72,73,74,75]. This observation may help prioritize TG (10:0)-related metabolism for future investigation, but it should not be interpreted as a validated acute stress marker without independent validation.

Candidate remodeling pattern of special structural modifications: Parallel evaluation of special structural linkages provided refined tracking of pathological responses (Figure 6B). Within the Cer subclass, deconstructed sphingoid bases (including d18:0 and d18:1) consistently exhibited higher relative abundance levels in both the AMI and PRMI groups relative to the healthy controls. This sub-molecular elevation directly complements our intact-lipid pathway mapping, providing structural evidence of accelerated sphingolipid mobilization under myocardial stress. Clinical data have firmly established that specific d18:1 sphingoid base-containing ceramides (such as Cer d18:1/16:0, Cer d18:1/24:1, and Cer d18:1/24:0) serve as independent biomarkers for fatal outcomes in cardiovascular patients [29], while dynamic fluctuations in d18:1/d18:0 sphingolipid backbones (including sphingosine-1-phosphate, S1P) are intimately intertwined with ischemic injury progression [32,76,77]. Mechanistically, the accumulation of these long-chain sphingoid bases determines functional specificity, directly orchestrating cardiac lipotoxicity by triggering mitochondrial dysfunction, disrupting organelle configurations like mitochondrial-associated membranes (MAM), and activating caspase-dependent apoptotic pathways in cardiomyocytes [30,78,79]. These deleterious cascades are governed by serine palmitoyltransferase long chain base subunit 2 (SPTLC2), the rate-limiting enzyme driving the d18:0 to d18:1 base desaturation and de novo synthesis track; its overactivation promotes lipotoxic cell death and sterile inflammation, whereas its targeted downregulation or suppression effectively blunts ceramide accumulation, mitigates apoptosis, and preserves post-infarction ventricular geometry [31,32].

Concurrently, deconstructed ether-linked chains (designated with e or p suffixes) within PC, PE, and LysoPC subclasses displayed highly distinct, subclass-specific variations (Figure 6B). Because vinyl-ether bonds render plasmalogens highly sensitive to oxidative stress, these specialized ether phospholipids act as essential endogenous antioxidant buffers that scavenge free radicals and safeguard membranes against ferroptotic lipid peroxidation [80,81]. Under pathological hypoxia, 15-lipoxygenase (15-LOX) can specifically target and oxidize plasmalogens into pathogenic hydroperoxy derivatives, accelerating structural loss [81]. The biogenesis of these crucial ether lipid species is strictly restricted to peroxisomes, which work in tight coupling with mitochondria to support respiratory chain supercomplex assembly and fine-tune reactive oxygen species (ROS) emissions [82,83,84]. Consequently, chronic hypoxia or overactivation of hypoxia-inducible factors triggers a severe loss of myocardial peroxisomes and a breakdown of ether lipid homeostasis, causing lines-of-defense collapse, line-of-respiration ROS bursts, and a profound hypersensitivity to ischemic injury that mirrors human heart failure and limits functional myocardial regeneration and repair mediated via PEX3-Akt axes [85,86]. These structured sub-molecular footprints offer robust, structure-weighted candidates for future biophysical validation.

### 4.4. Significance and Limitations

The core contribution of this study is not only the identification of broad lipid metabolic disturbances during MI and its recurrence, but also the presentation of a systematic, structure-oriented lipidomics analysis framework, LipiDecipher. Conceptually, it aims to move lipidomics analysis from the cataloguing of differential molecules toward more interpretable structure-associated biological hypotheses. Technically, by combining multi-dimensional feature prioritization with knowledge-based lipid–protein–pathway contextualization and structurally resolved analysis, it provides a practical framework for organizing complex lipidomic signals into biologically interpretable patterns.

Several limitations should be acknowledged. First, the deconstructed fatty acyl abundances generated by LipiDecipher represent mathematically deconstructed virtual remodeling indices based on mass-conservation principles, rather than the absolute physical concentrations of free fatty acids. While this class-specific projection is highly informative for tracing systemic lipid remodeling, it remains a semi-quantitative computational inference that relies on the accuracy of the parent lipid semi-quantification. Second, to prevent mathematical artifacts and annotation errors, our current pipeline applied a rigid filter excluding acyl chains with carbon numbers greater than 27. Consequently, ultra-long-chain fatty acid remodeling dynamics outside this range were not captured in this study. Third, although sex-disaggregated reporting and covariate-adjusted sensitivity analyses were added in the revised manuscript, the study groups were not perfectly balanced in sex composition, mainly because the HC group contained very few female participants (*n* = 2). The major lipidomic effect directions were largely preserved after adjustment for sex and age, but residual confounding cannot be fully excluded. To strictly evaluate the statistical boundaries imposed by our sample sizes, a formal post hoc power analysis was systematically performed using the pwr package in R. The results explicitly confirmed that the localized female-stratified baseline contrast (*n* = 2 in HC versus *n* = 10 in PRMI) suffered from severe under-powering (15.49% at a large effect size of Cohen’s d = 0.80). Consequently, consistent with this physical boundary, sex-stratified estimates involving female controls must be interpreted with extreme caution and treated as strictly baseline exploratory trends rather than definitive biomarker associations. Fourth, BMI data were not available in the baseline dataset and therefore could not be included as an additional covariate. Finally, the moderate sample size and single-cohort design underscore the need for validation in larger, more diverse, and prospectively collected cohorts. Nonetheless, our post hoc validation verified that for the global univariate screening across the three analytical arms (average *n* = 45 per group), the cohort achieved a substantial statistical power of 73.15% for capturing medium effect sizes (f = 0.25) and an exceptionally robust power of 98.92% for large effect sizes (f = 0.40) at a significance level (α) of 0.05. Furthermore, pairwise contrasts between the smaller clinical arms, specifically PRMI (*n* = 35) versus AMI (*n* = 50), maintained an excellent statistical power of 94.82% for detecting prominent phenotypic variations (d = 0.80), confirming that the primary cross-sectional lipidomic shifts captured by our framework remain statistically dependable within this study collective.

## 5. Conclusions

By applying the LipiDecipher analytical framework, this study delineated multi-level lipid remodeling patterns associated with the onset and recurrence of myocardial infarction and organized lipid structural changes into biologically relevant, database-supported metabolic contexts. The proposed framework illustrates its utility for handling complex lipidomics data, prioritizing candidate lipid signatures, and generating biologically plausible hypotheses from structure-oriented analyses. As an open-source and extensible tool, LipiDecipher may support future clinical lipidomics research, although external validation and experimental follow-up remain necessary before mechanistic or clinical applications can be established. The R code for the LipiDecipher framework is publicly available on GitHub at: https://github.com/AaronHwang8720/LipiDecipher (accessed on 7 July 2026).

## Figures and Tables

**Table 1:** Baseline characteristics of the study cohort.

Characteristic	HC (*n* = 50)	AMI (*n* = 50)	PRMI (*n* = 35)	*p* Value
Age, years	63.6 ± 12.4	65.2 ± 13.6	64.6 ± 12.0	0.808
Female, n (%)	2 (4.0%)	14 (28.0%)	10 (28.6%)	0.001

**Table 2:** Mapping of Fuzzy C-Means (FCM) cluster numbers to dynamic trajectory patterns and candidate biological implications.

Cluster 1	V-Shaped/Transient Decline	Decreased Sharply During Acute Onset (AMI) but Exhibited a Partial or Adaptive Recovery During Recurrence (PRMI).	Phospholipids (PC, PE)	Cellular Membrane Compensation
Cluster 2	Infarction-Specific/Transient Peak	Exhibited an acute and profound upregulation during AMI, followed by a substantial decline back toward baseline in PRMI.	Diacylglycerols (DG)	Acute-Phase Metabolic Stress
Cluster 3	Sustained/Progressive Upregulation	Elevated significantly during acute onset and remained high or amplified further during post-PCI recurrence.	Triglycerides (TG), Phosphatidylcholines (PC)	Continuous Remodeling & Overload
Cluster 4	Gradual/Mild Downward	Displayed a continuous, milder downward trend across the progressive spectrum of myocardial injury.	Mixed Glycerophospholipids	Chronic Structural Atrophy
Cluster 5	Sustained/Progressive Downregulation	Suffered a severe and permanent downregulation immediately after disease onset, remaining suppressed in recurrence.	Ceramides (Cer), Triglycerides (TG)	Impaired Hydrolysis & Signaling

## Data Availability

The R code used to implement the LipiDecipher framework is publicly available on GitHub at: https://github.com/AaronHwang8720/LipiDecipher (accessed on 7 July 2026). The processed lipidomics data underlying the main findings of this study, including lipid annotation metadata and major derived analysis outputs, are provided in the Supplementary Tables or are available from the corresponding author upon reasonable request. The raw mass spectrometry files are not publicly available at this stage due to institutional data-management restrictions and ongoing considerations regarding controlled data release. Access to such raw files may be considered on a case-by-case basis, subject to approval by the corresponding author, the relevant institution, and applicable ethical requirements.

## Supplementary Materials

The following supporting information can be downloaded at: https://www.mdpi.com/article/10.3390/metabo16070494/s1, Figure S1: Quality control assessment of the untargeted lipidomics dataset.; Figure S2. Covariate-adjusted sensitivity analysis and global intact lipidomic expression profiling.; Figure S3. Resampling-based feature stability assessment for category-specific LDA.; Figure S4. Repeated stratified 5-fold cross-validated performance of category-specific LDA.; Figure S5. Permutation-based validation of cross-validated LDA performance.; Figure S6. Apparent confusion matrices of category-specific LDA models.; Figure S7. Class-specific sub-molecular deconstructed acyl and alkyl chain remodeling heatmaps.; Table S1. Pairwise comparisons of baseline demographic characteristics across study groups.; Table S2. Sex-stratified summaries of major lipid features.; Table S3. Summary statistics of covariate-adjusted sensitivity analyses.; Table S4. Mass spectrometry metadata and MSI structural taxonomy for all analyzed lipid features.; Table S5. Molecular specifications and dosing concentrations of stable isotope-labeled internal standards.; Table S6. LDA coefficients for all lipid features.; Table S7. Top LDA contributors used in Figure 3.; Table S8. Lipid–protein–pathway contextualization collapsed at the lipid level.

## Abbreviations

Gene Ontology (GO), Kyoto Encyclopedia of Genes and Genomes (KEGG), Healthy Controls (HC), myocardial infarction (MI), Acute Myocardial Infarction (AMI), Post-PCI Recurrent Myocardial Infarction (PRMI), Quality Control (QC), Glycerolipids (GL), Glycerophospholipids (GP), Triacylglycerols (TG), Phosphatidylcholines (PC), Phosphatidylethanolamines (PE), Sterol lipids (ST), Sphingolipids (SP), Diacylglycerol (DG), Phosphatidylinositols (PI), Linear Discriminant Analysis (LDA), Sphingomyelins (SM), Ceramides (Cer), Cholesterol esters (ChE), Fuzzy C-Means (FCM), Lysophosphatidylcholines (LysoPC), Principal Component Analysis (PCA),one-way analysis of variance (ANOVA).
