# Supplementary material for: LipiDecipher: A Structure-Oriented Analytical Framework for Interpretable Clinical Lipidomics
Source: Metabolites. 2026 Jul 13;16(7):494. doi: 10.3390/metabo16070494 (PMC13414093; doi:10.3390/metabo16070494)
Supplement: Supplementary file 1 [file metabolites-16-00494-s001.zip › Supplementary_Figure S1- S7.pdf]

Supplemental figures

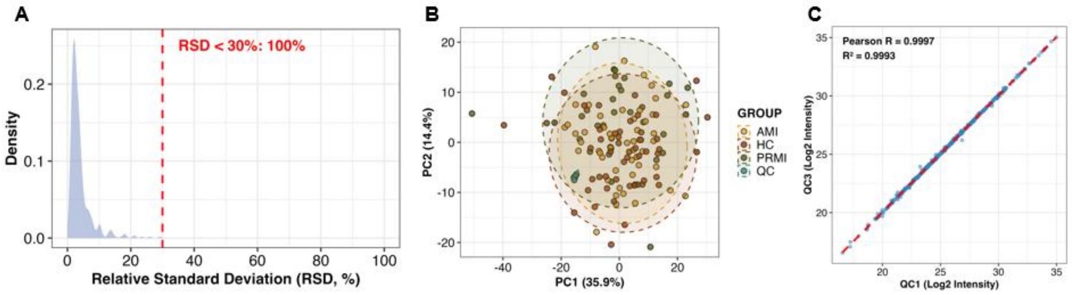

Supplementary Figure S1. Quality control assessment of the untargeted lipidomics dataset. (A) Principal component analysis (PCA) of QC and biological samples showing the clustering pattern of pooled QC injections relative to study samples. (B) Distribution of relative standard deviation (RSD) values across lipid features in QC samples. Features with excessive missingness or QC RSD >30% were removed during preprocessing. (C) Correlation analysis between representative QC injections QC1 and QC3. The high correlation between QC injections supports analytical reproducibility across the acquisition batch.

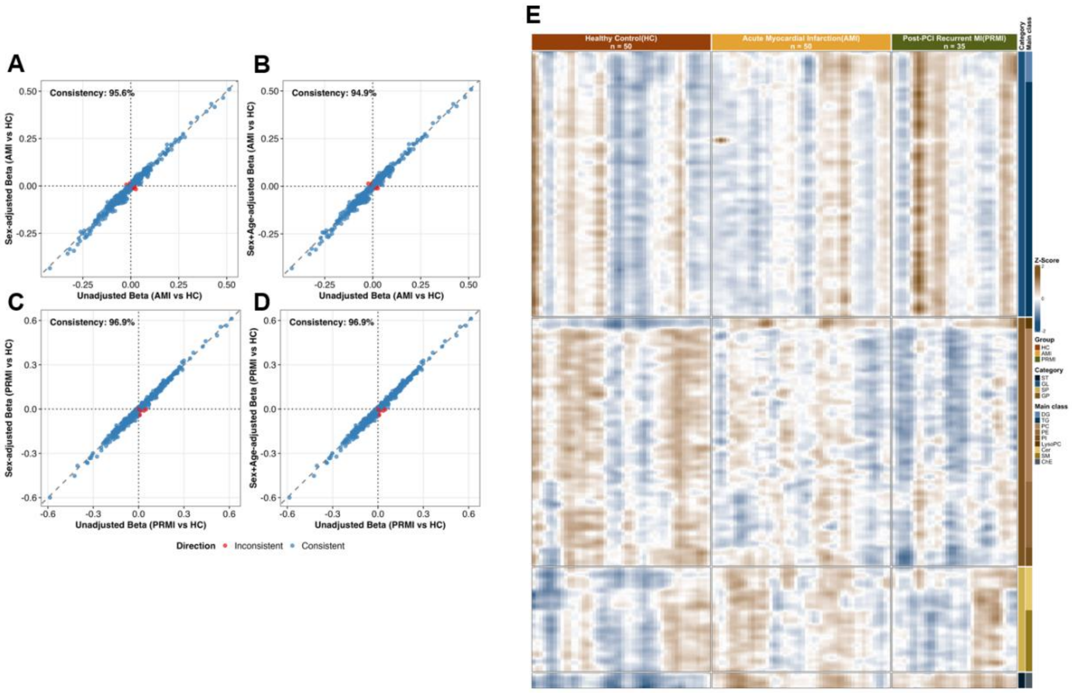

Supplementary Figure S2. Covariate-adjusted sensitivity analysis and global intact lipidomic expression profiling. (A–D) Scatter plots comparing the standardized group-effect coefficients (Beta estimates) derived from unadjusted baseline linear models (X-axis) against those derived from multi-covariate sensitivity-adjusted models (Y-axis). Panels (A) and (B) illustrate the AMI vs. HC group contrast adjusted for sex alone (A) or sex plus age simultaneously (B). Panels (C) and (D) illustrate the PRMI vs. HC group contrast adjusted for sex alone (C) or sex plus age simultaneously (D). Each individual point projects a single monitored lipid feature, and the diagonal dashed line represents the line of perfect equivalence (Y = X). The exceptionally high directional consistency across all four panels demonstrates that the cross-sectional lipidomic alterations are fundamentally driven by myocardial infarction pathology rather than baseline age or sex imbalances. (E) Hierarchical clustering heatmap capturing the global cross-sectional expression landscape of the intact lipidomic profiles across the independent healthy control (HC), acute myocardial infarction (AMI), and post-PCI recurrent myocardial infarction (PRMI) patient arms.

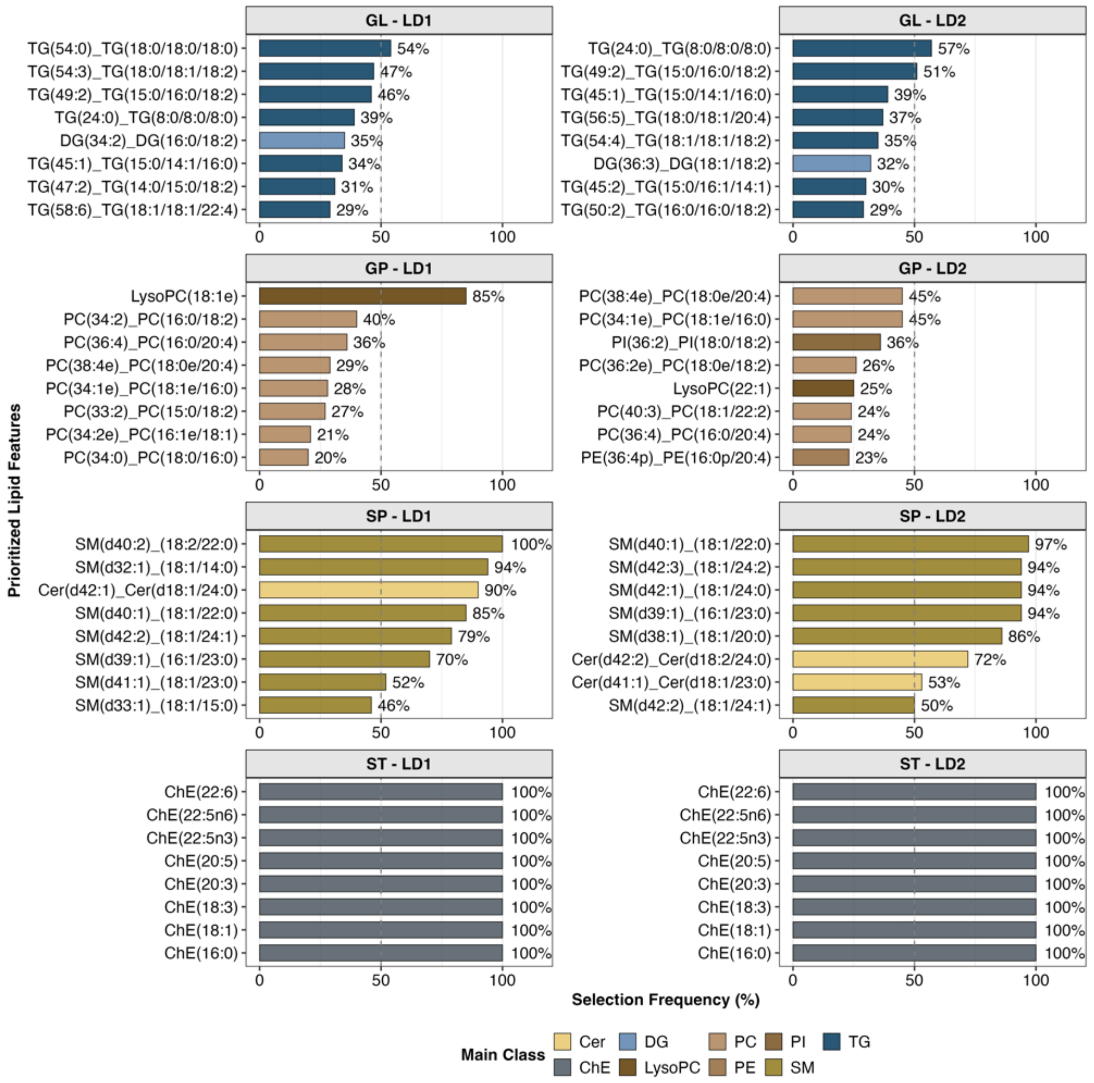

Supplementary Figure S3. Resampling-based feature stability assessment for category-specific LDA.

Feature stability was evaluated using 100 iterations of stratified 80% subsampling. In each iteration, top lipid contributors along LD1 and LD2 were recorded. Bars indicate the selection frequency of lipid features across resampling iterations. Higher selection frequency indicates more stable contribution to within-cohort discriminant structure. These results assess feature-level robustness under sample perturbation and do not constitute external validation.

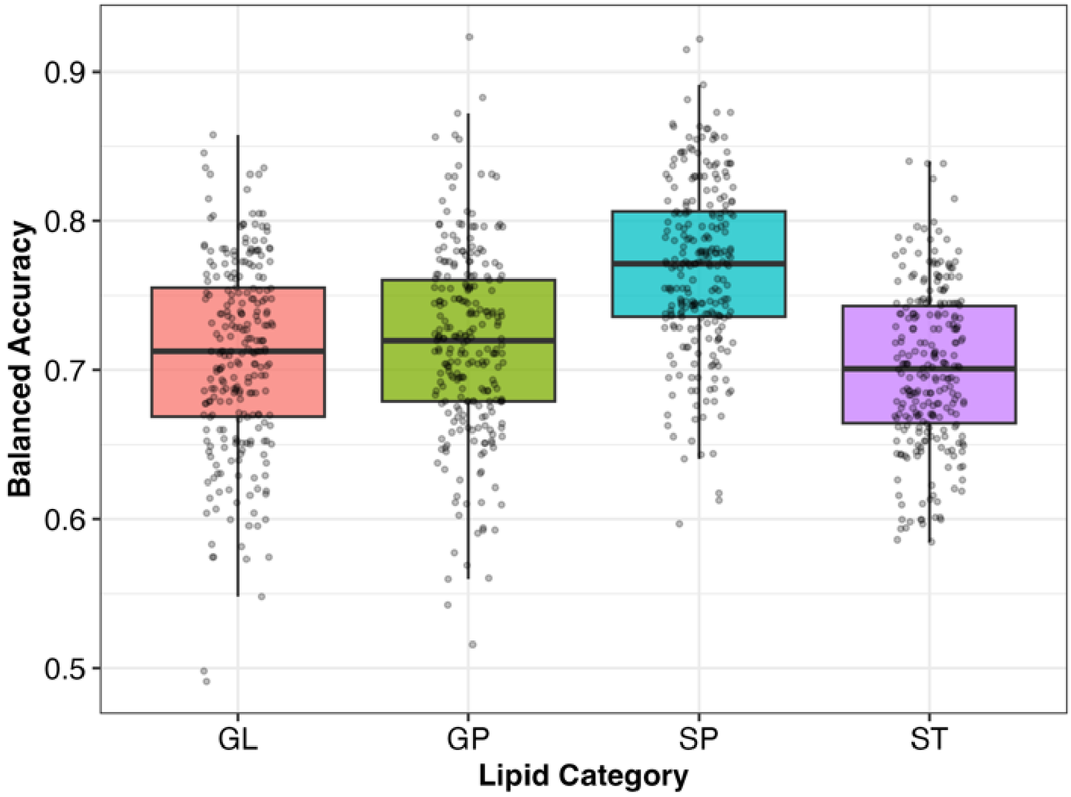

Supplementary Figure S4. Repeated stratified 5-fold cross-validated performance of category-specific LDA.

Category-specific LDA models were evaluated using repeated stratified 5-fold cross-validation. For each fold, models were trained on the training subset and evaluated on the held-out subset. Boxplots summarize the distribution of balanced accuracy across cross-validation repeats and folds for each lipid category. These results provide internal model-level performance estimates and should not be interpreted as validation in an independent external cohort.

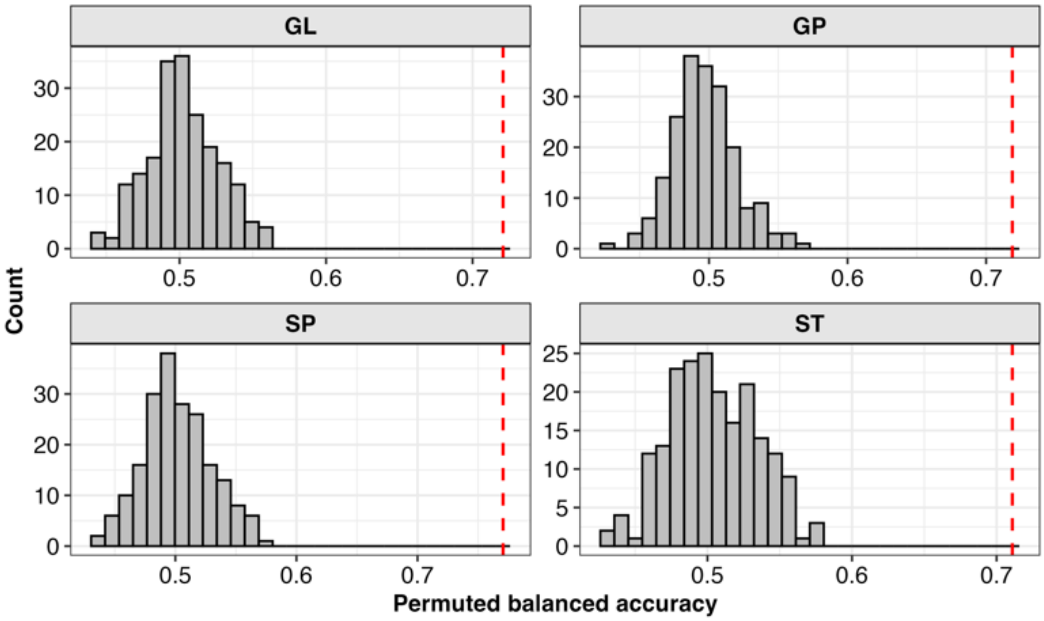

Supplementary Figure S5. Permutation-based validation of cross-validated LDA performance. Histograms show null distributions of balanced accuracy generated from 200 random label permutations. Red dashed lines indicate the observed balanced accuracy obtained under true group labels using repeated stratified 5-fold cross-validation. No permuted model exceeded the observed performance in any lipid category, yielding permutation $P < 0.005$. These results indicate that the observed category-specific discriminant patterns were unlikely to arise from random label structure, although they remain internal validation results.

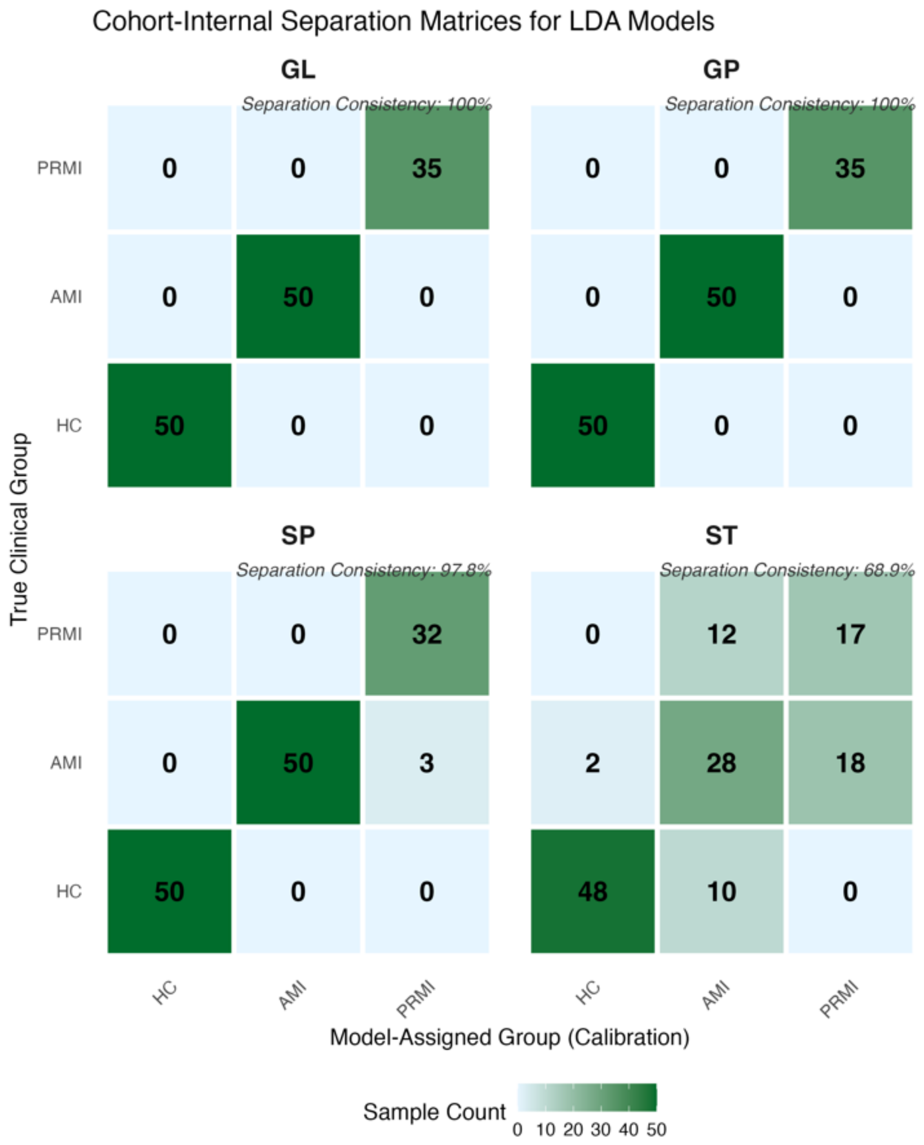

Supplementary Figure S6. Apparent confusion matrices of category-specific LDA models. These matrices represent apparent classification performance obtained by fitting and predicting within the full dataset. They are provided for descriptive visualization of within-cohort group separation only. They should not be interpreted as external validation or unbiased estimates of clinical classifier performance. Model-level robustness was evaluated separately using repeated cross-validation and permutation testing, as shown in Supplementary Figures S4 and S5.

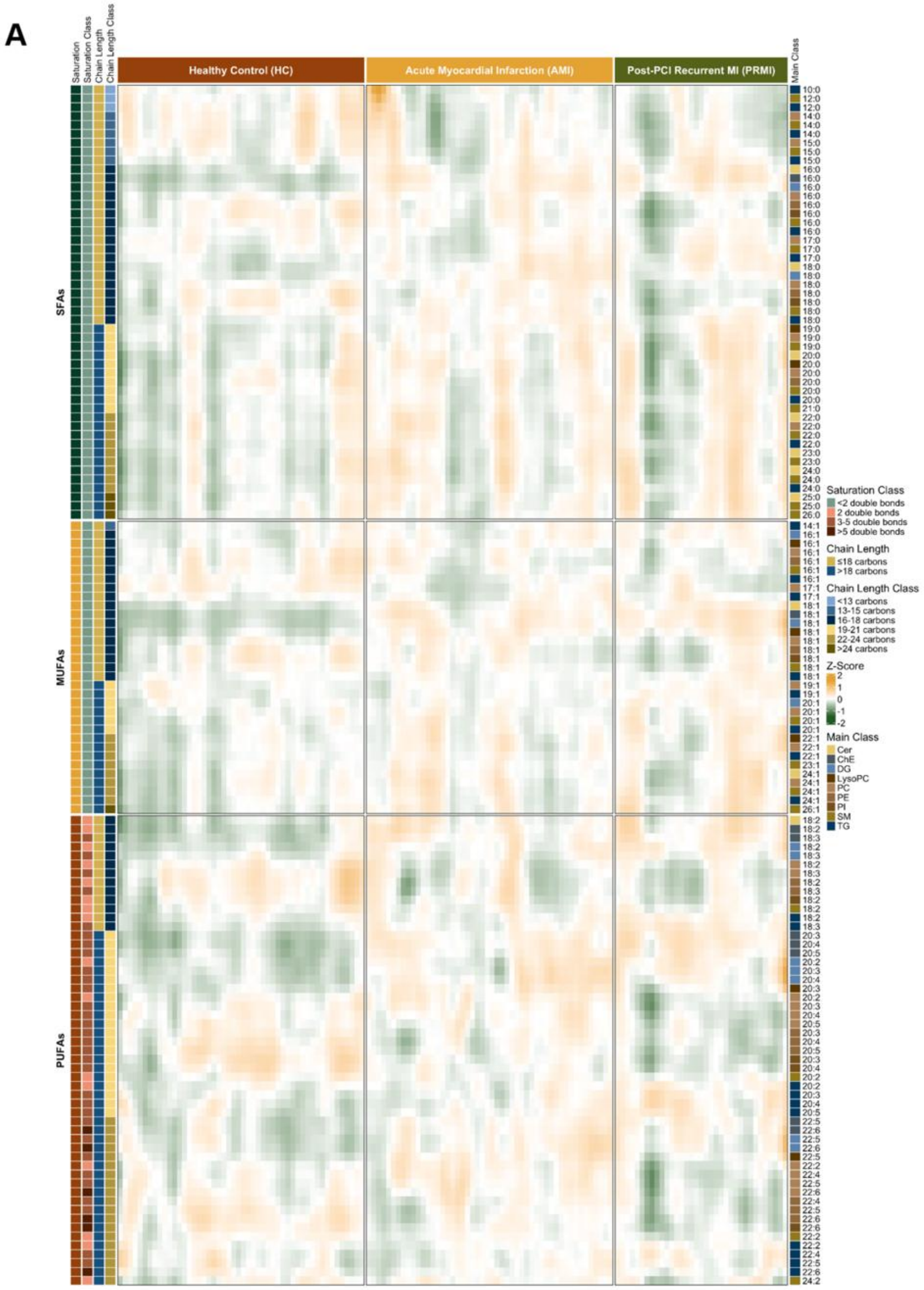

Supplementary Figure S7. Class-specific sub-molecular deconstructed acyl and alkyl chain remodeling heatmaps.

Hierarchical clustering heatmaps visualizing the virtual abundance trends of deconstructed sub-molecular components across the study cohorts, derived by processing intact lipid measurements through the molecular-weight-proportional attribution (MWP) algorithm. The profiles illustrate cross-sectional group differences at the individual fatty acyl and ether-linked alkyl/plasmalogen chain levels within specific major lipid main classes. These matrices summarize localized structural remodeling trends and trace systematic shifts in chain-length distributions and unsaturation degrees across independent clinical arms, providing structural context to augment intact-molecular findings.
