# Supplementary material for: LipiDecipher: A Structure-Oriented Analytical Framework for Interpretable Clinical Lipidomics
Source: Metabolites. 2026 Jul 13;16(7):494. doi: 10.3390/metabo16070494 (PMC13414093; doi:10.3390/metabo16070494)
Supplement: Supplementary file 1 [file metabolites-16-00494-s001.zip › Supplementary_STROBE-checklist-v4-combined.pdf]

STROBE Statement—checklist of items that should be included in reports of observational studies

	Item No	Recommendation
**Title and abstract**	1	(a) Indicate the study's design with a commonly used term in the title or the abstract<br>The abstract explicitly indicates the study design as a "retrospective serum lipidomics dataset" and a "retrospective acute myocardial infarction cohort".
		(b) Provide in the abstract an informative and balanced summary of what was done and what was found<br>The abstract provides a balanced summary of the background, analytical framework development, major structural lipid alterations identified, and cautious, knowledge-based conclusions.
**Introduction**		
Background/rationale	2	Explain the scientific background and rationale for the investigation being reported<br>The Background explains the clinical potential of lipidomics, the bottleneck in structure-function interpretation using current generic workflows, and the rationale for a structure-oriented approach.
Objectives	3	State specific objectives, including any prespecified hypotheses<br>The study aims to develop "LipiDecipher", a modular analytical framework to navigate complex lipidomics data and prioritize structure-associated biological hypotheses.<br>The study aims to develop "LipiDecipher", a modular analytical framework to navigate complex lipidomics data and prioritize structure-associated biological hypotheses.
**Methods**		
Study design	4	Present key elements of study design early in the paper<br>Described early in the Methods, the study is designed as a retrospective clinical cohort comprising three groups: healthy controls (HC), acute myocardial infarction (AMI), and post-PCI recurrent MI (PRMI).
Setting	5	Describe the setting, locations, and relevant dates, including periods of recruitment, exposure, follow-up, and data collection<br>The study setting was the First Affiliated Hospital of Dalian Medical University, and the retrospective cohort samples along with clinical data were collected in April 2022.
Participants	6	(*a*) *Cohort study*—Give the eligibility criteria, and the sources and methods of selection of participants. Describe methods of follow-up<br>*Case-control study*—Give the eligibility criteria, and the sources and methods of case ascertainment and control selection. Give the rationale for the choice of cases and controls<br>*Cross-sectional study*—Give the eligibility criteria, and the sources and methods of selection of participants<br>Participants were classified into specific cohorts (HC, AMI, PRMI) based on clinical diagnosis and event recurrence.
		(*b*) *Cohort study*—For matched studies, give matching criteria and number of exposed and unexposed<br>*Case-control study*—For matched studies, give matching criteria and the number of controls per case

Variables	7	Clearly define all outcomes, exposures, predictors, potential confounders, and effect modifiers. Give diagnostic criteria, if applicable<br>The primary exposures/phenotypes are the clinical transition stages. Sex and age were defined and assessed as potential confounders.
Data sources/ measurement	8*	For each variable of interest, give sources of data and details of methods of assessment (measurement). Describe comparability of assessment methods if there is more than one group<br>Lipid variables were assessed via a modified MTBE extraction protocol and UHPLC-HRMS, with structures identified following MSI standards.
Bias	9	Describe any efforts to address potential sources of bias<br>Bias was addressed through covariate-adjusted sensitivity analyses (for sex and age), strict multi-stage analytical filtering (CV<30%), and internal validation (permutation testing, cross-validation).
Study size	10	Explain how the study size was arrived at<br>Due to the retrospective nature, an a priori calculation was not performed; however, a systematic post-hoc power analysis (via R pwr package) confirmed robust statistical power for ANOVA (98.92%) and pairwise testing.
Quantitative variables	11	Explain how quantitative variables were handled in the analyses. If applicable, describe which groupings were chosen and why<br>Quantitative lipid peak areas were normalized, kNN-imputed, and mathematically deconstructed into mass-proportional virtual sub-molecular remodeling indices.
Statistical methods	12	(a) Describe all statistical methods, including those used to control for confounding<br>Statistical methods included global ANOVA, Dunnett's post-hoc tests, systematic Benjamini-Hochberg FDR correction, Fuzzy C-Means clustering, and linear regression models adjusted for sex and age.
		(*b*) Describe any methods used to examine subgroups and interactions
		(*c*) Explain how missing data were addressed
		(*d*) *Cohort study*—If applicable, explain how loss to follow-up was addressed<br>*Case-control study*—If applicable, explain how matching of cases and controls was addressed<br>*Cross-sectional study*—If applicable, describe analytical methods taking account of sampling strategy
		(*e*) Describe any sensitivity analyses

Continued on next page

**Results**		
Participants	13*	(a) Report numbers of individuals at each stage of study—eg numbers potentially eligible, examined for eligibility, confirmed eligible, included in the study, completing follow-up, and analysed<br>The final analyzed study cohort included 50 HC, 50 AMI, and 35 PRMI individuals (total N=135).
		(b) Give reasons for non-participation at each stage
		(c) Consider use of a flow diagram
Descriptive data	14*	(a) Give characteristics of study participants (eg demographic, clinical, social) and information on exposures and potential confounders<br>Baseline demographic and clinical characteristics (age, sex) are detailed in Table 1 and Supplementary Table S1.
		(b) Indicate number of participants with missing data for each variable of interest
		(c) *Cohort study*—Summarise follow-up time (eg, average and total amount)
Outcome data	15*	*Cohort study*—Report numbers of outcome events or summary measures over time
		Case-control study—Report numbers in each exposure category, or summary measures of exposure
		Cross-sectional study—Report numbers of outcome events or summary measures<br>For this retrospective cohort, the summary measures of exposure and outcome (lipidomic feature peak areas and relative fold changes across clinical transitions) are thoroughly reported.
Main results	16	(a) Give unadjusted estimates and, if applicable, confounder-adjusted estimates and their precision (eg, 95% confidence interval). Make clear which confounders were adjusted for and why they were included<br>Unadjusted models and confounder-adjusted sensitivity analyses (adjusted for sex and age) were performed. Directional consistency between unadjusted and adjusted estimates was evaluated.
		(b) Report category boundaries when continuous variables were categorized<br>Not applicable. Continuous variables (lipid normalized peak areas) were not categorized into groups.
		(c) If relevant, consider translating estimates of relative risk into absolute risk for a meaningful time period<br>Not applicable. This is a molecular lipidomics study, not a traditional epidemiological risk assessment.
Other analyses	17	Report other analyses done—eg analyses of subgroups and interactions, and sensitivity analyses<br>Supplementary analyses included structure-abundance linear correlations, resampling-based feature stability assessments, repeated cross-validation, permutation testing, and sex-stratified exploratory trends.
**Discussion**		
Key results	18	Summarise key results with reference to study objectives<br>The LipiDecipher framework successfully identified MI-associated lipid remodeling patterns and organized complex sub-molecular variations into database-supported, biologically plausible hypotheses, achieving the study's primary objective.
Limitations	19	Discuss limitations of the study, taking into account sources of potential bias or imprecision. Discuss both direction and magnitude of any potential bias

		Acknowledged limitations include the semi-quantitative nature of the virtual remodeling indices, exclusion of >27 carbon chains, absence of BMI data, and the severely under-powered female-stratified baseline contrast limiting generalizability.
Interpretation	20	Give a cautious overall interpretation of results considering objectives, limitations, multiplicity of analyses, results from similar studies, and other relevant evidence<br>Results are interpreted with extreme epistemic caution; pathway mappings and enzyme-related proxies are explicitly framed as descriptive, database-supported hypotheses rather than direct causal or functional evidence.
Generalisability	21	Discuss the generalisability (external validity) of the study results<br>The authors explicitly note that as a retrospective single-cohort study, independent validation in larger, more diverse, and prospective cohorts is required before mechanistic or generalizable clinical claims can be established.
**Other information**		
Funding	22	Give the source of funding and the role of the funders for the present study and, if applicable, for the original study on which the present article is based<br>Sources of funding are explicitly disclosed, including the Young Elite Scientists Sponsorship Program by CAST, and Science and Technology Plans of Liaoning Province.

*Give information separately for cases and controls in case-control studies and, if applicable, for exposed and unexposed groups in cohort and cross-sectional studies.

Note: An Explanation and Elaboration article discusses each checklist item and gives methodological background and published examples of transparent reporting. The STROBE checklist is best used in conjunction with this article (freely available on the Web sites of PLoS Medicine at http://www.plosmedicine.org/, Annals of Internal Medicine at http://www.annals.org/, and Epidemiology at http://www.epidem.com/). Information on the STROBE Initiative is available at www.strobe-statement.org.
